# Efficacy and Safety of Stapled Transanal Rectal Resection (STARR) for Obstructed Defecation Syndrome: A Systematic Review and Meta-Analysis of Comparative Studies

**DOI:** 10.3390/jcm15197347

**Published:** 2026-09-22

**Authors:** Khaled S. Abbas, Walid M. Abd El Maksoud, Fahad S. Al Amri, Hassan A. Alzahrani, Abdullah Dalboh, Marei H. Alshandeer, Maha A. Alghamdi, Haytham M. Fayed, Ahmad Sakr, Ahmad Jebril M. Bosaily, Khaled Elshaar, Mohammed A. Bawahab

**Affiliations:** 1 Department of General Surgery, College of Medicine, King Khalid University, Abha 61421, Saudi Arabia; khassan@kku.edu.sa (K.S.A.); fsalamri@kku.edu.sa (F.S.A.A.); hahassan@kku.edu.sa (H.A.A.); dalboh@kku.edu.sa (A.D.); ac4473@kku.edu.sa (M.H.A.); malamodi@kku.edu.sa (M.A.A.); hfayed@kku.edu.sa (H.M.F.); asakr@kku.edu.sa (A.S.); ajmbosaily@kku.edu.sa (A.J.M.B.); mbawahab@kku.edu.sa (M.A.B.); 2 Department of General Surgery, Jazan General Hospital, Jazan Health Cluster, Jazan 82723, Saudi Arabia; kali-al-shaar@moh.gov.sa

**Keywords:** obstructed defecation syndrome, stapled transanal rectal resection (STARR), constipation, postoperative morbidity, systematic review and meta-analysis

## Abstract

**Background/Objectives:** Stapled transanal rectal resection (STARR), introduced in 2004 for obstructed defecation syndrome (ODS), remains of uncertain comparative efficacy and safety relative to other therapeutic approaches. This study systematically evaluated and meta-analyzed STARR surgery versus alternative interventions for ODS. **Methods:** MEDLINE (PubMed), CENTRAL, ProQuest, SAGE, and Web of Science were searched up to May 2026 for randomized controlled trials and observational comparative studies evaluating STARR against any comparator for ODS. Primary outcomes included improvement in validated ODS constipation scores and postoperative morbidity (bleeding, pain, urgency/incontinence). Secondary outcomes were operation time and hospital length of stay. Pooled effect sizes were calculated using random-effects models, heterogeneity assessed via I^2^ statistics, and cumulative meta-analyses performed to examine temporal trends. **Results:** Twelve studies (4 RCTs, 8 observational) comprising 995 participants were included. No significant difference in ODS score improvement was found between STARR and comparators (SMD = 0.26, 95% CI: −0.17 to 0.70; I^2^ = 89.2%); however, due to substantial clinical and statistical heterogeneity and wide prediction intervals, this pooled estimate reflects an average of diverse underlying effects rather than a single definitive treatment effect with cumulative analysis revealing loss of initial efficacy after 2010. STARR showed numerically higher but non-significant odds of bleeding (OR = 1.73) and urgency/incontinence (OR = 2.91), with comparable postoperative pain (OR = 0.70). STARR demonstrated significantly shorter operation time (MD = −21.23 min) and hospital stay (MD = −1.15 days). **Conclusions:** STARR surgery offers no superior efficacy in ODS symptom improvement compared with alternative treatments. However, the extreme heterogeneity of the primary outcome limits the interpretive value of the pooled estimates. While there are numerically higher odds of postoperative bleeding and urgency/incontinence, these differences did not reach statistical significance. STARR is associated with significantly shorter operation time and hospital stay. The declining effect size over time and substantial heterogeneity suggest that patient selection is critical. Future large multicenter RCTs with long-term follow-up and standardized patient-reported outcomes are needed to define optimal surgical strategies for specific ODS phenotypes.

## 1. Introduction

Chronic constipation is a highly prevalent functional gastrointestinal disorder characterized by difficult, infrequent, or perceived incomplete evacuation of bowel contents, affecting approximately 9–20% of the general population, with consistently higher prevalence among women and older adults [1,2]. It is broadly classified into primary (functional) and secondary forms, with primary constipation encompassing three distinct subtypes: normal-transit constipation, slow-transit constipation, and defecatory disorders [3,4]. Secondary constipation arises from identifiable systemic or structural causes, including medication side effects, metabolic and endocrine disorders, neurological diseases, and structural gastrointestinal abnormalities [3,5]. Understanding this classification is fundamental to directing appropriate diagnostic workup and selecting the most effective therapeutic strategy for each patient.

Diagnosis relies on the Rome IV criteria, which require at least two characteristic symptoms persisting for a minimum of six months, providing a standardized framework for distinguishing functional constipation from organic etiologies and other functional bowel disorders [1,4]. Chronic constipation is consistently associated with significant impairment of health-related quality of life and markedly increased healthcare utilization, imposing a substantial burden on patients, caregivers, and healthcare systems worldwide [5,6,7].

Obstructed Defecation Syndrome (ODS) is a clinically distinct subtype of chronic constipation defined by impaired rectal evacuation due to functional and/or structural anorectal abnormalities [5]. Population-based studies report a prevalence of ODS in the range of 3–10% in the general adult population, though rates as high as 20–30% have been observed depending on diagnostic criteria and symptom definitions [8,9]. ODS is estimated to account for approximately 30–50% of all chronic constipation cases, yet it remains frequently underdiagnosed due to substantial overlap with other functional bowel disorders [8,9]. The condition disproportionately affects middle-aged women, particularly those who are multiparous, have a history of obstetric pelvic floor injury, or have undergone urogynecological surgery [7]. Cardinal symptoms include excessive straining, fragmented or incomplete stool passage, a sensation of pelvic heaviness, prolonged defecation time, and the need for manual assistance to facilitate evacuation [5,7]. Beyond its physical impact, ODS imposes a substantial psychological and social burden, with significant adverse effects on quality of life, sexual function, and daily activities [9].

The etiology of ODS involves a combination of mechanical and functional anorectal derangements. Mechanical contributors include rectocele, internal rectal prolapse, and intussusception, while functional causes encompass dyssynergic defecation and impaired rectal sensation [5,7]. Diagnosis requires a multimodal approach incorporating colonoscopy, or CT colonography, anorectal manometry, transrectal ultrasonography, and defecography [7,9]. Disease severity is objectively quantified using the validated Obstructed Defecation Syndrome score (ODS score), an eight-item instrument widely used in clinical practice and research to stage severity and monitor treatment response [10]. Given the complexity of its etiology, management of functional ODS necessitates a multidisciplinary approach involving psychologists, neurologists, physiatrists, and surgeons, while mechanical pathologies, namely rectocele, internal prolapse, and intussusception, are primarily addressed through surgical correction [9,11].

The management of ODS encompasses a spectrum of therapeutic strategies. Conservative measures, including dietary modification, osmotic and stimulant laxatives, pelvic floor physiotherapy, and biofeedback therapy, constitute the first-line approach [5,11]. Biofeedback, which targets dyssynergic defecation through neuromuscular retraining, has demonstrated efficacy specifically in functional ODS but offers limited benefit in patients with predominant mechanical pathology [12]. When conservative measures fail, surgical options are considered, including modified Delorme’s procedure, laparoscopic ventral mesh rectopexy (LVMR), and transvaginal rectocele repair [5,11]. Each surgical approach carries distinct indications, technical considerations, and outcome profiles, and no single intervention has been established as the definitive standard of care for all ODS presentations.

Stapled Transanal Rectal Resection (STARR) was introduced in 2004 as a minimally invasive transanal technique designed to address the mechanical component of ODS through endorectal stapled resection of the anterior and posterior walls of the distal rectum, thereby simultaneously correcting rectocele and internal rectal intussusception [13]. Two principal variants have since been developed: the original double-stapler technique using the PPH-01 circular stapler and the Contour Transtar technique employing a curved cutter stapler that permits a larger and more circumferentially tailored resection [14,15]. Early multicenter prospective data demonstrated high rates of symptomatic improvement, driving its widespread adoption. However, variability in patient selection criteria, particularly regarding the degree and combination of rectoceles and intussusception, has been proposed as a key determinant of outcomes and a principal source of heterogeneity across published series [11].

Despite initial enthusiasm, subsequent evidence raised substantive concerns regarding the long-term efficacy, recurrence rates, and safety profile of STARR, most notably the risks of postoperative urgency, rectal bleeding, staple-line complications, and de novo fecal incontinence, particularly compared with alternative therapeutic approaches [2,11]. Prior systematic reviews have assessed the efficacy and safety of individual therapeutic approaches for ODS using single-arm analyses; most notably, Ripamonti et al. [7] reported generally favorable symptom relief outcomes following STARR, but their analysis was confined exclusively to non-comparative studies, thereby precluding any conclusions regarding the relative efficacy and safety of STARR versus alternative interventions. To date, no meta-analysis of comparative studies has synthesized this evidence.

The present study, therefore, aims to systematically review and meta-analyze all available comparative studies evaluating the efficacy and safety of STARR versus other therapeutic modalities, including biofeedback therapy, laparoscopic ventral mesh rectopexy, the Contour Transtar technique, and conservative medical therapy, for the treatment of ODS, with respect to symptom improvement, functional outcomes, patient satisfaction, and perioperative and long-term complication rates.

## 2. Materials and Methods

This meta-analysis pooled the efficacy and safety of STARR surgery among patients with ODS. We performed this systematic review and meta-analysis according to the Preferred Reporting Items of the Systematic Reviews and Meta-Analyses (PRISMA) guidelines and the Cochrane Handbook for Systematic Reviews of Interventions 2023 [16]. The research protocol was registered in PROSPERO (CRD420261382519).

Website: https://www.crd.york.ac.uk/PROSPERO/view/CRD420261382519, accessed on 2 May 2026.

### 2.1. Criteria for Considering Studies

We included randomized/non-randomized clinical trials and observational comparative studies assessing the beneficial effects of the treatments. We excluded cross-sectional studies, case reports, and case series. We searched for studies published only in English, with no publication date restriction.

Regarding the population, we included studies on adult patients of both sexes with ODS due to mechanical or functional reasons. Studies involving other colorectal diseases were excluded.

The intervention was surgical treatment of ODS with STARR. The comparators were either (1) standard conservative treatment (e.g., laxatives) or (2) other surgical corrective procedures for ODS, including Laparoscopic Ventral Mesh Rectopexy (LVMR), Single Stapled Trans-Anal Prolapsectomy with Perineal Levatorplasty (STAPL), Contour Transtar, Delorme operation, or Bresler operation.

### 2.2. The Outcomes and Definitions

#### 2.2.1. Primary Outcomes 

Treatment success was measured by standardized mean differences (SMD) in validated ODS scoring systems either by the Obstructed Defecation Syndrome score (ODS score), modified ODS, Cleveland Constipation Score (CCCS), Altomare ODS, or Wexner Constipation Score.

Postoperative morbidity measured by pooled OR of persistent postoperative complications after 1 month from surgery, including:

Bleeding;
Pain;
Urgency and/or incontinence.

#### 2.2.2. Secondary Outcomes 

Were the Differences in the Operation Time (Minutes) and Hospital Length of Stay (Days).

### 2.3. Data Sources and Search Strategy

We conducted a comprehensive literature search in May 2026 by two independent authors (K. A. & W. A.) to identify all published and unpublished studies in the English language without publication date or geographical restrictions. We searched MEDLINE (PubMed), CENTRAL (The Cochrane Central Register of Controlled Trials), ProQuest, SAGE, and Web of Science (WOS). Highly sensitive search strategies were developed by H. F. after approval of the PubMed Help Desk. Potential search terms are (“stapled transanal rectal resection” OR “laparoscopic ventral rectopexy” OR “internal Delorme” OR “contour TranStar”) AND (“obstructive defecation syndrome” OR defecation OR “intestinal obstruction” OR “rectal prolapse” OR intussusception) AND (outcome OR “postoperative complication*”). Supplementary File S1 shows a detailed search strategy for each database, along with appropriate search terms.

### 2.4. Study Selection and Eligibility Criteria

Two independent reviewers (F. A. & A. S.) screened titles and abstracts for all retrieved studies to relevant ones. We excluded all non-comparative studies and all studies that did not include STARR in comparison. Then, two authors (A.D. & H.A.) independently assessed the retrieved abstracts and the full texts of these studies, guided by the Cochrane form for data extraction, to formulate a table of characteristics of included studies. In both phases of screening, the reviewers handled disagreements through consultation with a third reviewer (M.A.). To minimize publication bias and identify additional eligible studies not captured by electronic searches, we performed backward citation tracking by screening the reference lists of all full-text articles assessed for eligibility and all included studies. The extracted data included study characteristics (first author, publication year, study design, and sample size), participant characteristics (mean age, gender, diagnosis), surgical properties (comparator name, length of stay, operation time, and ODS scoring system), and outcomes (primary outcomes and secondary outcomes).

### 2.5. Assessment of Methodological Quality of Included Studies

Randomized controlled trials were assessed through the Cochrane Risk-of-Bias tool (ROB2). It assesses different types of bias in five domains. We judged risk of bias criteria as ‘low risk,’ ‘high risk,’ or ‘unclear risk’ and evaluated individual bias items as described in the Cochrane Handbook for Systematic Reviews of Interventions [16]. We summarize the overall quality of the meta-analysis included studies according to ROB2 into high risk, some concerns, or low risk. The observational studies were assessed using the Newcastle–Ottawa Scale for quality evaluation of non-randomized studies [17]. It assessed the quality in three domains. The quality score was either low risk/good (7–10 points), some concern/fair (3–6 points), or high risk/poor (0–2 points). Finally, we presented a ‘risk of bias summary’ figure for each assessment tool.

### 2.6. Statistical Analysis Plan

The statistical analyses were conducted using R 4.4.3 software. For continuous outcome data, effect size was calculated as MD/SMD using Hedges’ g method, while categorical outcomes were calculated through OR using the Mantel–Haenszel method. Differences were considered statistically significant at *p* < 0.05. Forest plots were used to show individual studies and meta-analysis estimates in a RevMan style. Cumulative meta-analyses were conducted sequentially by ordering studies chronologically to evaluate the evolution of pooled effect estimates (ORs, MDs, or SMDs) and their precision as accumulated evidence. We estimated the magnitude of the effect according to Cohen’s d scale. A fixed-effects model meta-analysis was performed, but if the Cochrane Q test *p*-value < 0.1 and Higgins’ I^2^ > 50% indicated a significant heterogeneity between studies, we performed a random-effects model.

Heterogeneity was assessed visually in the forest plot, predicted interval, and Higgins’ I^2^ statistic, which was interpreted as follows: 0% to 40%: might not be important; 30% to 60%: may represent moderate heterogeneity; 50% to 90%: may represent substantial heterogeneity; and 75% to 100%: considerable heterogeneity. To explain the substantial heterogeneity, we conducted several sensitivity analyses: a leave-one-out analysis to assess the influence of individual studies on the pooled effect estimate. Baujat plots with influence diagnostics were used to identify outlier studies contributing disproportionately to heterogeneity or the overall effect.

The publication bias was assessed through visual inspection of the funnel plot, trim and fill plot, Egger’s test for outcomes that include at least ten studies, and Begg’s test for outcomes that include fewer than ten studies.

## 3. Results

### 3.1. Search Results

A total of 822 were searched for potentially relevant articles. After removal of duplications (149 by EndNote, four manually), the studies screened by title and abstract for relevance were 669. We excluded 637 irrelevant articles, and only 32 articles were eligible for full-text screening. Five studies could not be retrieved; qualitative analyses included 27 articles. One study did not include data for ODS score change, four were inappropriate study designs, 10 had irrelevant objectives, and one was applied to mice (Figure 1). Backward citation tracking added one study to the included list.

### 3.2. Quality Assessment

#### 3.2.1. Characteristics of the Studies

We included 12 studies in the meta-analysis, five from Italy [11,15,18,19,20] and seven distributed in Germany [14], France [21,22], the United States of America [23], Iran [24], China [25], and Egypt [26]. The total count of participants was 499 in the STARR branch with a mean age of 55.7 ± 6.9 and 94.5% of them were females and 496 in the control branch with a mean age of 57.9 ± 6.4 and 93.0% females. The cause of ODS among patients was rectocele, rectal intussusception, and rectal prolapse. Four studies were RCTs [11,19,22,26], while the rest studies were observational [14,15,18,20,21,23,24,25]. Table 1 shows the summary of narrative synthesis.

#### 3.2.2. Quality Assessment Results

For observational studies, two studies were of good quality [15,25], and six studies had fair quality [14,18,20,21,23,24]. For RCTs, all studies are low quality and have a high risk of bias (Supplementary Figure S1A,B).

### 3.3. Outcomes

#### Mean Improvement in ODS Constipation Scores

This objective included twelve studies with a total of 970 participants (Madbouly et al. [26] has smaller number of participants calculated the mean change in ODS score (46 in experimental arm and 41 in controls while 56 in each arm in the rest of objectives)). The pooled SMD of ODS score was statistically insignificant at 0.26 (95% CI of −0.17; 0.70). This means that the STARR surgical approach has a comparable effect with controls on improved ODS scores (Figure 2A). This null finding could be interpreted with extreme statistical heterogeneity (I^2^ = 89.2%, *p* < 0.0001), which explores dramatic variation across studies, evidenced by the prediction interval (−1.22, 1.75). Given the extreme statistical heterogeneity (I^2^ = 89.2%) and the wide prediction interval, the pooled SMD provides limited interpretive value as a single overall effect. In accordance with current methodological guidance for meta-analyses with substantial clinical and methodological diversity, this random-effects estimate reflects the average of a wide range of underlying treatment effects rather than a single definitive outcome. Therefore, the interpretation of the primary outcome relies more heavily on the cumulative meta-analysis and subgroup evaluations to understand the temporal and clinical evolution of STARR’s efficacy. Cumulative meta-analyses were conducted to illustrate how the effect size evolves over the number of studies included. It showed the initial efficacy of STARR surgery in the treatment of ODS with the clinical and statistical significance of SMD. Inclusion of the fifth study in 2011 was associated with loss of statistical significance and a shift toward unfavorable point estimates throughout the rest of evidence. The in-between study heterogeneity rose to exceed 80% during the same period, suggesting that methodological or clinical variability introduced after 2010 substantially influenced the pooled effect and undermined the early promise of STARR for ODS (Figure 2B).

Lehur et al. [22], Isbert et al. [14] and Madbouly et al. [26] were identified as outliers (Supplementary Figure S2A). These studies exhibited extreme effect sizes (SMD =1.61, 1.08, and −0.62 respectively), far exceeding the range observed in other trials (SMD: −0.40 to 1.04). The leave-one-out sensitivity analysis confirmed their influence on the pooled estimate, where the pooled SMD after excluding Lehur et al. [22] was 0.14 (95% CI: −0.24; 0.5, I^2^ = 84.5%), after excluding Isbert et al. [14] was 0.18 (95% CI −0.26; 0.63, I^2^ = 87.3%), and after exclusion of Madbouly et al. was 0.35 (95% CI −0.09; 0.79, I^2^ = 87.9%) (Supplementary Figure S2B).

The funnel plot (Supplementary Figure S2C) showed asymmetrical distribution of studies, with potential aggregation of points on the right side indicating potential publication bias or small-study effects. However, Egger’s test was not significant (t = −0.52, *p* = 0.62)).

### 3.4. Postoperative Complications

#### 3.4.1. Pain

Postoperative pain was assessed among 520 participants (262 in the STARR arm and 258 controls) in six studies [11,14,19,21,25,26] using a random-effects model (Figure 3A). The individual OR ranged from 0.44 to 1.36 with an insignificant pooled estimate and consistent homogeneity of results across all included studies (OR = 0.70, 95% CI: 0.36; 1.36, I^2^ = 0%). The earliest studies suggested a minor protective effect with wide confidence intervals, indicating substantial insignificance and imprecision. By inclusion of more recent studies, the cumulative effect estimate remained around the null, confirming a statistically insignificant association (Figure 3B). The analysis of different types of surgery (Contour-Transtar, LVR, and others) showed similar results in all groups. Specifically, the pooled estimates within subgroups remained non-significant: Contour-Transtar (OR = 0.94, 95% CI: 0.22–4.08), LVR (OR = 0.54, 95% CI: 0.15–1.91), and other techniques (OR = 0.71, 95% CI: 0.28–1.82). There was no evidence of a subgroup effect (χ^2^ = 0.33, df = 2, *p* = 0.8483), suggesting that the type of comparator surgery had no significant impact on the treatment effect (Figure 3C).

Publication bias was assessed using a funnel plot (Supplementary Figure S2D) and Begg’s rank correlation test; both tools excluded heterogeneity, with symmetric results for the funnel plot and non-significant results (Kendall’s τ = −0.333, *p* = 0.4694).

#### 3.4.2. Bleeding

Nine studies that reported bleeding events included 751 participants. The pooled OR supports higher odds of bleeding in the STARR arm more than in controls with absent between-study heterogeneity (OR = 1.73, 95% CI: 0.88; 3.39, I^2^ = 0%). In the cumulative meta-analysis, the pooled OR shifted away from the null during the sequential addition of the first four studies (from 1.00 (0.06; 16.93) to 2.18 (0.73; 6.55)), with precision improving as evidence accumulated. Early cumulative estimates were imprecise and not statistically significant, and between the fifth and seventh studies the effect remained only marginally non-significant (OR 1.94–2.17; *p* = 0.065–0.097). The cumulative estimate reached statistical significance solely after the addition of the eighth study (OR 2.13 (1.02; 4.45), *p* = 0.043); however, this significance was transient; the addition of the final study [25] attenuated the estimate toward the null, yielding a final non-significant pooled OR of 1.73 (95% CI 0.88–3.39; *p* = 0.111). Thus, the apparent benefit of STARR suggested by the earlier cumulative data was not sustained as evidence accumulated, and the overall conclusion of no statistically significant difference between STARR and control rests on the most complete and precise data (Figure 4). Across all subgroups, confidence intervals were wide, reflecting imprecision. For studies using “Other” surgical techniques, the pooled effect estimate was OR = 1.48 (95% CI: 0.62–3.54). Similarly, comparisons with Contour-Transtar showed an OR of 2.28 (95% CI: 0.55–9.45), while LVR comparisons yielded an OR of 4.00 (95% CI: 0.43–37.14). The single study comparing STARR with the Delorme operation also demonstrated a non-significant effect (OR = 0.50, 95% CI: 0.03–8.77).

The funnel plot (Supplementary Figure S2E) showed potential publication bias and small study effects. However, Egger’s test could not reject the null hypothesis (t = −0.49, *p* = 0.641).

#### 3.4.3. Stool Urge and/or Incontinence

The pooled OR of urge/incontinence was assessed in 556 patients in eight studies conducted between 2004 and 2018. OR was 2.91 (95% CI: 0.99; 8.55) (Figure 5A). Despite the OR value indicating nearly a threefold increase in risk, the cumulative analysis could not reach statistical significance for the association in all time periods (Figure 5B). Leave-one-out sensitivity analysis identified Savastano et al. [15] as an influential study; exclusion of this study reduced heterogeneity to 36.9%. Subgrouping indicates comparable ORs of urge/incontinence against comparators (Figure 5C). Visual inspection of the funnel plot indicates slight asymmetry and a potential small study effect that is supported by trim-and-fill analysis (trim fill) (Supplementary Figure S2F–H). Begg’s test excluded the publication bias assumption (Kendall’s τ = 0.00, *p* = 1.00).

### 3.5. Secondary Outcomes

The pooled analysis shows a statistically significant reduction in operation time favoring the STARR (MD = −21.23, 95% CI: −39.84; −2.61). However, substantial heterogeneity was observed across the included studies (I^2^ = 99.1%, *p* < 0.0001), confirmed by the wide prediction interval (−79.30 to 36.85) (Figure 6). Based on the analysis in R, the studies by Madbouly et al. and Lian and Wang were considered influential for heterogeneity (Supplementary Figure S2I). Figure 7 shows a statistically significant reduction in hospital LOS in the STARR arm compared with controls (MD = −1.15 days, 95% CI: −1.90; −0.39). However, substantial heterogeneity was observed (I^2^ = 97.2%), and the wide prediction interval (−3.62 to 1.32) suggests considerable variability in the point estimate across studies. This heterogeneity could not be explained by leave-one-out sensitivity analysis. The Baujat plot identified Lian and Wang [25], Madbouly et al. [26], and Harris et al. [23] as contributors to heterogeneity (Supplementary Figure S2K,L).

For publication bias, the bias related to operation time was assessed visually and showed an aggregation of most studies on the left side of the diagonal line, indicating asymmetry in the funnel plot. However, Egger’s regression test did not provide statistical evidence of publication bias (t = −2.18 *p* = 0.656). For LOS, most studies aggregated the right side of the funnel plot. However, Egger’s regression test did not provide statistical evidence of publication bias (t = 0.49, *p* = 0.640) (Supplementary Figure S2J,M).

## 4. Discussion

ODS is a prevalent subtype of chronic constipation characterized by impaired rectal evacuation and associated with deterioration in general health and negative consequences for physical and psychological health. Despite the evolution of recent therapeutic and surgical approaches, management of ODS remains challenging. So, we aimed to assess the effectiveness and safety of STARR surgeries for ODS. The studies included 12 comparative studies on 995 patients classified between STARR surgery (499 patients) and controls (496 patients).

The analysis reported extreme heterogeneity in pooled efficacy estimates which provided a limited interpretive value. This heterogeneity is multifactorial and reflects the clinical diversity among comparator interventions that differ in surgical access, anatomical targets, degree of rectal wall resection, correction of pelvic floor support, and therapeutic philosophy. Therefore, pooling these interventions under a single comparator group may inherently generate variability in treatment effects.

Another major contributor to heterogeneity is the diversity of ODS phenotypes among included populations. ODS is not a single disease entity but rather a syndrome arising from multiple structural and functional abnormalities, including rectocele, rectal intussusception, internal prolapse, perineal descent, pelvic floor dyssynergia, and mixed pelvic floor disorders. Ripamonti et al. [7] concluded that STARR provides greater benefit in patients with predominant mechanical obstruction, whereas patients with functional pelvic floor dysfunction or colonic dysmotility may experience less long-term improvement. In our analysis, most of the included studies evaluated patients with mechanical ODS, except Lian and Wang [25] and Biviano et al. [18], who had mixed functional-mechanical ODS.

The pooled SMD of the ODS constipation score in the current study was 0.26 (95% CI of −0.17; 0.70). Despite being statistically insignificant, the forest plot initially shows an improvement in effect sizes, followed by a significant deterioration with the inclusion of studies published after 2010. This pattern may reflect differences in follow-up duration across studies. Follow-up periods varied substantially, from approximately 8 to 41 months, potentially affecting the assessment of sustained symptomatic benefit. Longer follow-up periods provide better assessment of the sustainability of the surgical outcomes and trajectory of ODS scores over time. Mandovra et al. [27] reported a substantial reduction in the Longo ODS score from 23 preoperatively to 7 immediately after STARR surgery, followed by a gradual increase up to 16 at 60 months, suggesting partial loss of long-term efficacy over time. Anderioli et al. [28] supported the same assumption. However, because the present cumulative meta-analysis was ordered by publication year rather than follow-up duration, and because follow-up duration was highly variable across studies, this explanation should be considered hypothesis-generating rather than causal. Moreover, variations in surgical strategies over time targeting improvement of postoperative outcomes for patients may contribute to decline of impact of STARR. Renzi et al. [29] utilized STARR combined with Transperineal Sonography (TPS) as a comparator vs. STARR alone. The recent technique led to shifting the mean difference in ODS score favoring the combination from 1.04 ± 2.43 to 7.13 ± 2.38. Hence, the acceptance of STARR in the treatment of ODS is decreasing among colorectal surgeons [30].

Compared to the comparator approaches, STARR was linked to statistically insignificant increase in bleeding odds (OR = 1.73, 95% CI: 0.88; 3.39) and urgency/incontinence (OR = 2.91, 95% CI: 0.99; 8.55). On the other hand, postoperative pain pooled OR was comparable between groups (OR = 0.70, 95% CI: 0.36;1.36). Regarding bleeding, the higher odds in STARR approach may reflect differences in the extent and configuration of rectal-wall resection, staple-line characteristics, vascular tissue involvement, and intraoperative hemostasis, as well as the number of staplers used. The comparator interventions were heterogeneous, ranging from alternative stapled procedures, such as (STAPL [11], TRANSTAR [19], Contour-Transtar [14,15], and Bresler operation [25], to non-stapled surgical approaches (Delorme surgery [20,24], LVR [21,26], TVR [23], biofeedback: [22], and conservative treatment [18]. Moreover, the risk of bleeding is attributed to the difficulty of managing high-vascularity segments when utilizing multiple staplers [30,31].

Most comparators—like STARR—involved rectal wall resection or stapled line reconstruction through stapling techniques or manual resection. Hence, the inflammatory response and healing cascade are assumed to be comparable in both arms [32]. Nevertheless, the magnitude of postoperative pain is a multifactorial outcome influenced by the location and extent of tissue trauma across procedures, as well as variation in perioperative analgesic protocols and the timing and definition of pain assessment [33]. Notably, the included studies did not provide sufficiently granular or standardized data regarding these confounding variables. In the absence of such information, a definitive judgment on the comparability of postoperative pain odds between STARR and its comparators cannot be drawn, and the pooled estimate should therefore be interpreted with caution. Further trials employing uniform pain assessment scales, standardized analgesic regimens, and consistent time-point evaluations are warranted to elucidate true between-group differences.

Similarly, STARR showed a trend toward increased postoperative urgency/incontinence compared with comparator procedures (OR = 2.91, 95% CI: 0.99–8.55), although the confidence interval included the null value. Several physiological mechanisms may potentially contribute to this finding. STARR surgery alters the rectal compliance and reservoir function through circumferential resection, leading to disturbance in rectal sensitivity and anorectal coordination and increasing urgency and incontinence symptoms [34,35]. Moreover, extensive stapled resection may affect rectal sensory pathways and lower the threshold for urgency perception [36,37]. These physiological alterations may explain persistent bowel dysfunction. In contrast, other techniques presented better performance postoperatively; LVR stabilizes the rectum and corrects internal prolapse without resecting rectal wall layers and restores physiological sensation [38]. Likely, Delorme operation involves mucosal stripping and plication with manual suturing while sparing the muscularis to maintain normal rectal sensory function [39]. However, differences in baseline disease severity, rectal prolapse characteristics, pre-existing continence impairment, and surgical technique may also contribute to the observed variation in postoperative urgency and incontinence across studies.

Nine studies reported a statistically significant reduction in operation duration and hospital LOS in the STARR arm compared to comparators. This may be attributed to its apparent efficiency, as a minimally invasive surgery avoids abdominal access and pelvic reconstruction, reduces anesthesia exposure time, and accelerates postoperative recovery [40]. For surgeons, it offers surgical benefits to counteract its long-term complications. From a health economic perspective, STARR offers lower costs of care and reduction in resource utilization when compared to equivalent open techniques [24,41]. Additionally, patients could return to their daily activities. Despite postoperative complications, Madbouly et al. [26] and Lian and Wang [25] reported higher postoperative satisfaction rates in STARR, underscoring the negative effect of ODS on quality of life before surgery [6]. Overall, despite postoperative complications, most patients reported satisfaction with the surgical outcome and perceived improvement in their quality of life following the procedure.

### Strengths and Limitations

The current study included several strengths. First, to the best of our knowledge, this is the first meta-analysis to assess the efficacy and safety of STARR surgery compared to other available surgical interventions. Second, including RCTs and observational studies reflects robust evidence for clinical decision-makers. Third, using various statistical analysis methods and incorporating cumulative meta-analysis offers chronological follow-up for efficacy and safety parameters of STARR in 20 years. However, the findings should be interpreted with caution due to limitations; first, unexplained heterogeneity in outcomes limits the confidence of pooled estimates; the extreme heterogeneity observed in the outcomes suggests that quantitative pooling may not be entirely appropriate, as the pooled estimate represents an average of highly diverse underlying effects rather than a uniform treatment benefit. Consequently, interpretation of e effect sizes should be taken into consideration. Second, total complication rates could not be pooled due to incomplete reporting. Third, the small number of studies included in some outcomes limits the precision and generalizability of the corresponding effect sizes.

## 5. Conclusions

STARR surgery offers no superior efficacy in ODS symptom improvement compared with alternative treatments. However, the extreme heterogeneity of the primary outcome limits the interpretive value of the pooled estimates. While there are numerically higher odds of postoperative bleeding and urgency/incontinence, these differences did not reach statistical significance. STARR is associated with significantly shorter operation time and hospital stay. The declining effect size over time and substantial heterogeneity suggest that patient selection is critical. Future large multicenter RCTs with long-term follow-up and standardized patient-reported outcomes are needed to define optimal surgical strategies for specific ODS phenotypes.

## Figures and Tables

**Figure 1:**
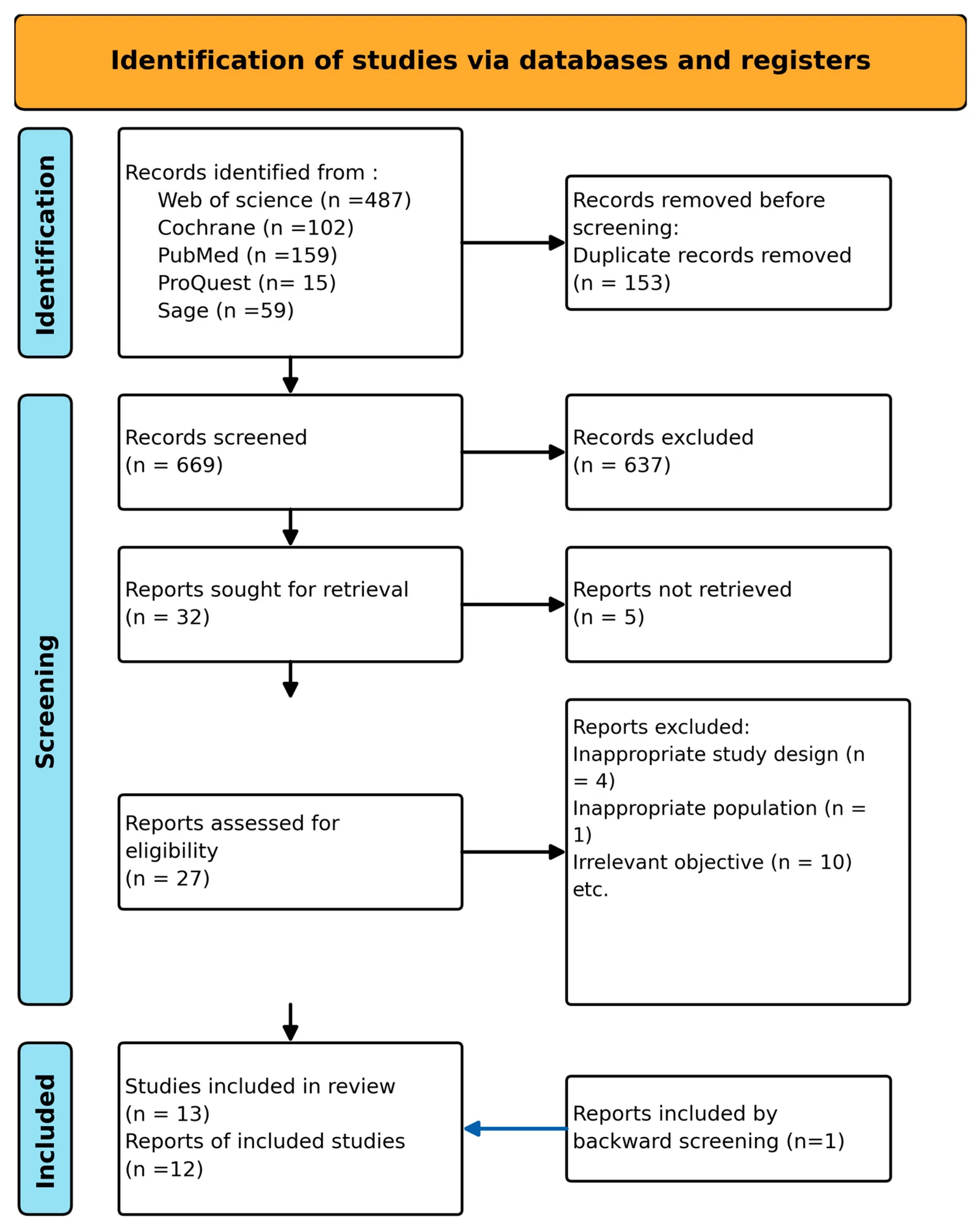
PRISMA flowcharts of studies included in the meta-analysis of efficacy and safety of STARR surgery for obstructive defecation syndrome.

**Figure 2:**
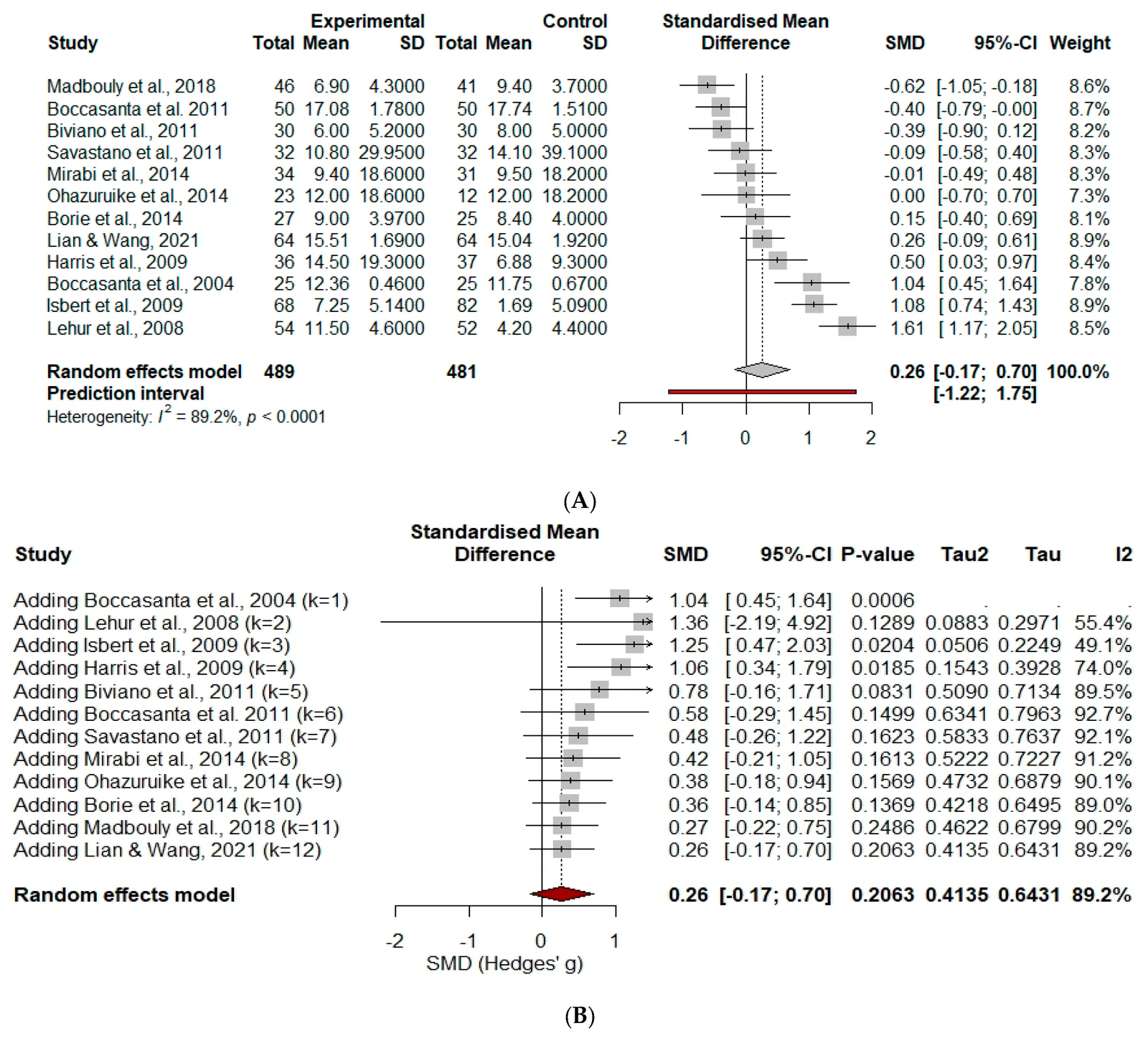
Forest plots comparing postoperative pooled change in ODS constipation scores between STARR and control management. (**A**) Conventional meta-analysis of ODS score change. (**B**) Cumulative meta-analysis of ODS score change [11,14,15,18,19,20,21,22,23,24,25,26].

**Figure 3:**
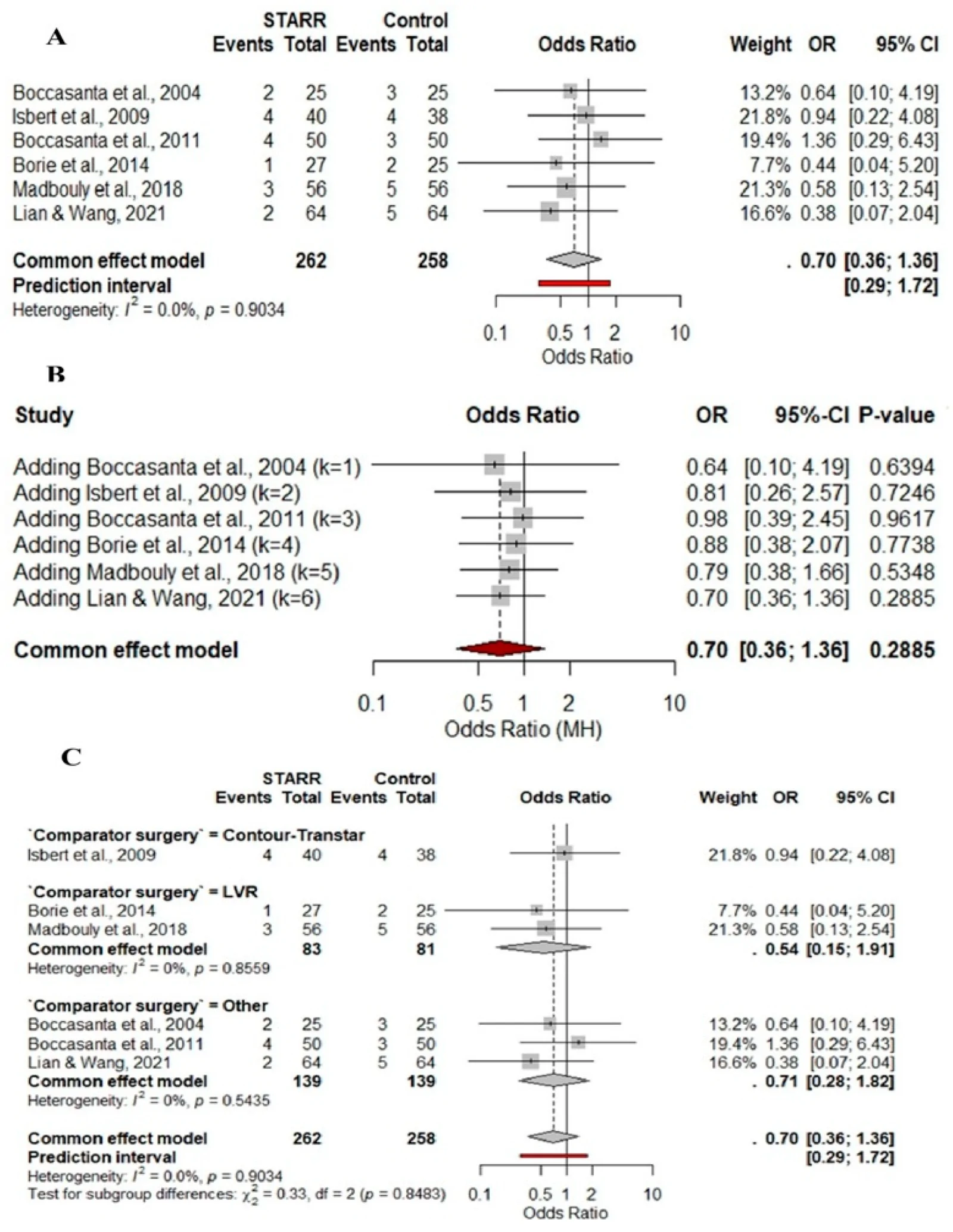
Shows forest plots of pooled odds ratios for postoperative pain in patients with ODS comparing STARR surgery with control management. (**A**) Conventional meta-analysis. (**B**) Cumulative meta-analysis. (**C**) Subgrouping by comparator surgery (others: STARR + TPS, STAPL, TRANSTAR) [11,14,19,21,25,26].

**Figure 4:**
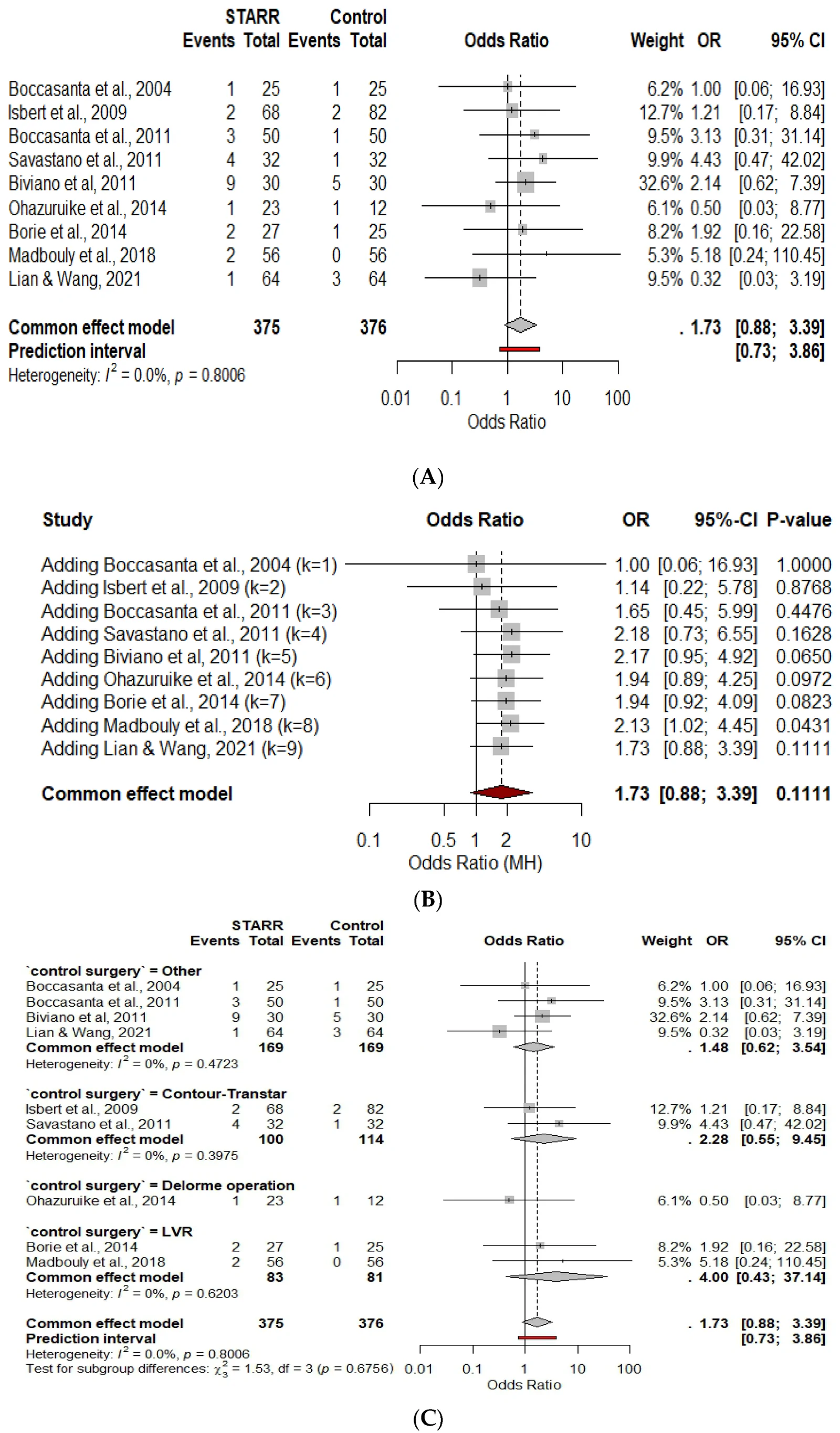
Shows forest plots of pooled odds ratios for postoperative bleeding in ODS patients comparing STARR surgery with control management. (**A**) Conventional meta-analysis. (**B**) Cumulative meta-analysis. (**C**) Subgrouping by comparator surgery (others: STARR + TPS, STAPL, TRANSTAR) [11,14,15,18,19,20,21,25,26].

**Figure 5:**
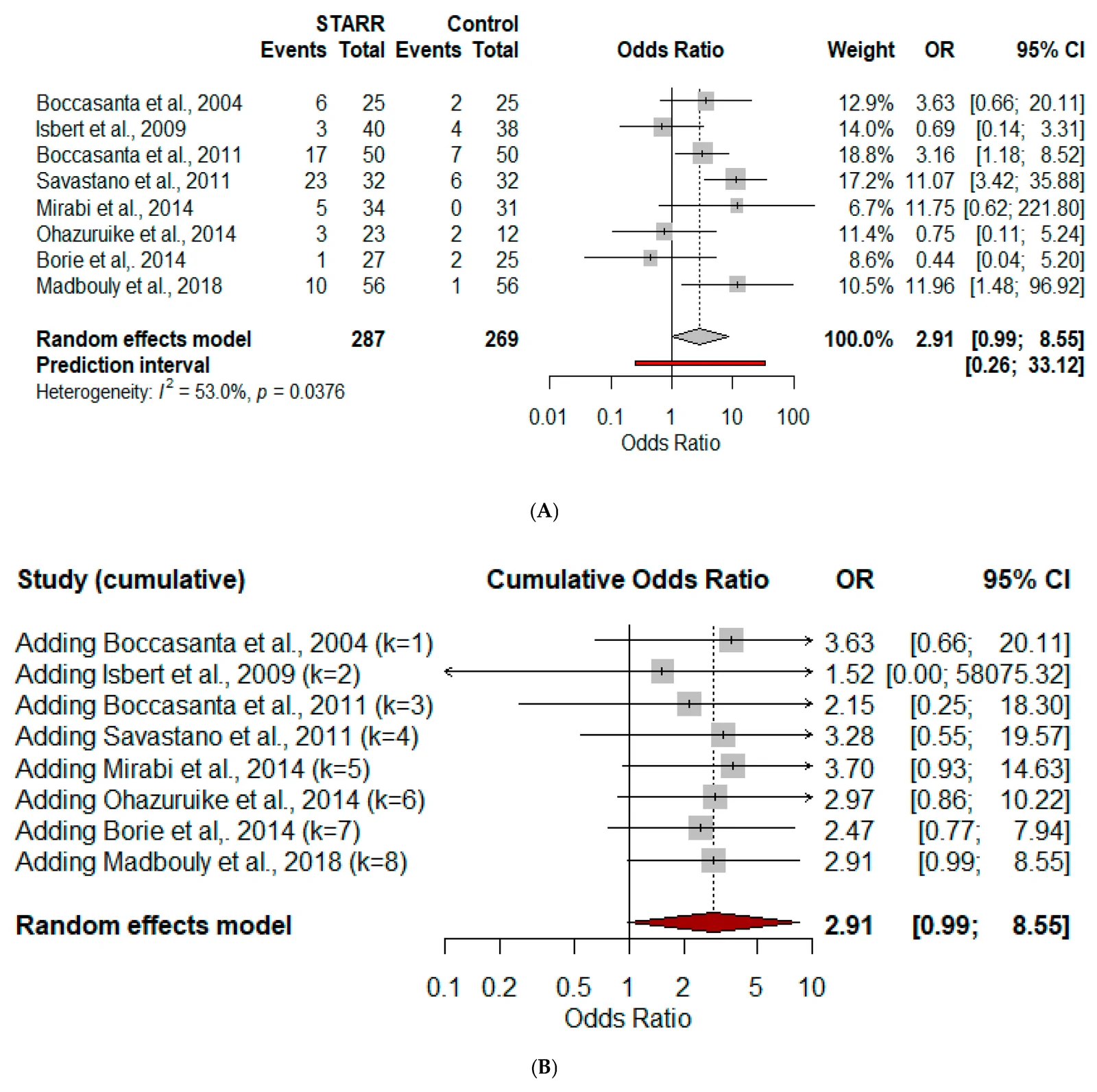

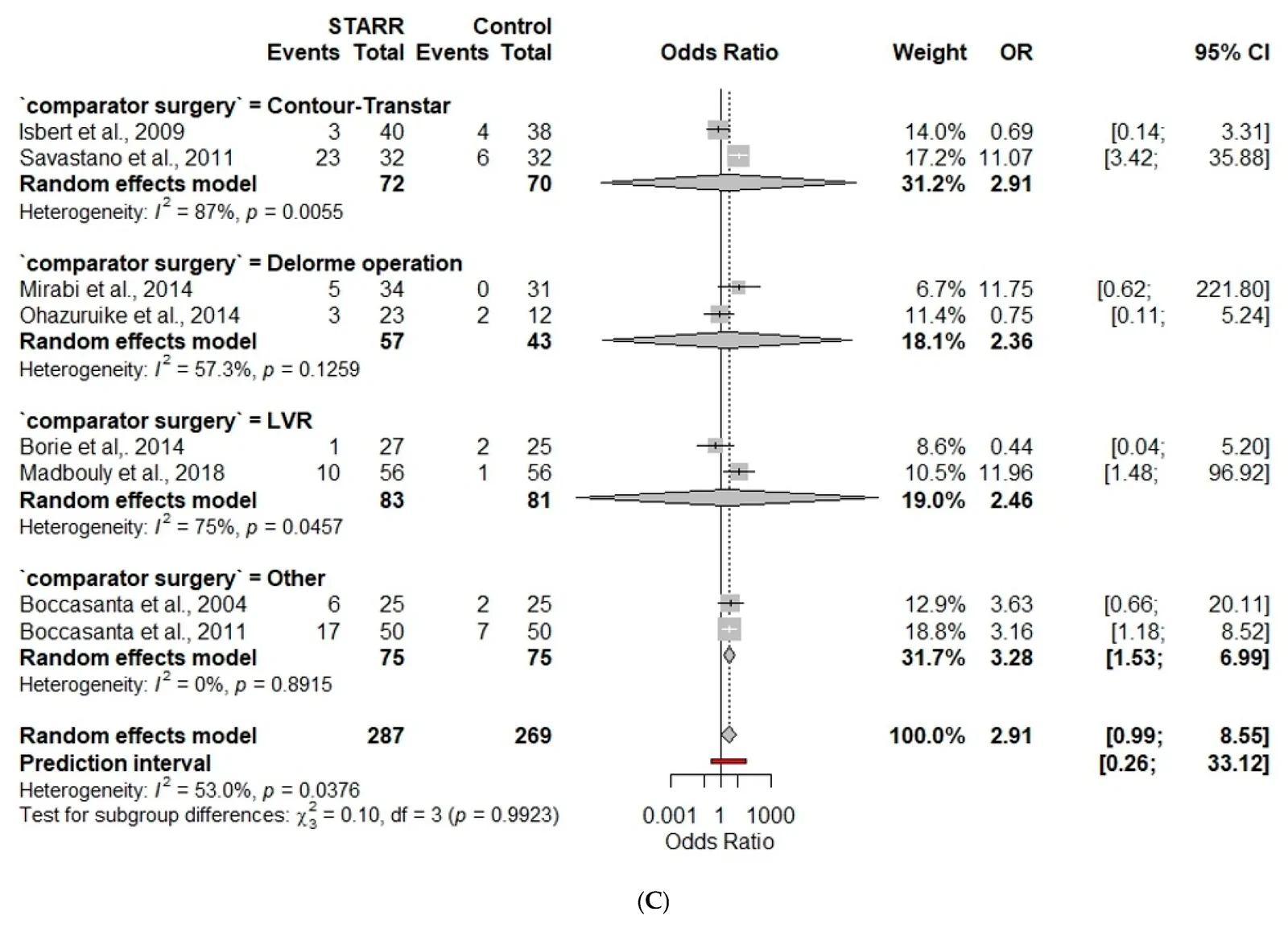
Forest plots of pooled odds ratios for postoperative urge/incontinence in ODS patients comparing STARR surgery with control management. (**A**) Conventional meta-analysis. (**B**) Cumulative meta-analysis. (**C**) Subgrouping by comparator surgery (Others: STARR + TPS, STAPL, TRANSTAR) [11,14,15,19,20,21,24,26].

**Figure 6:**
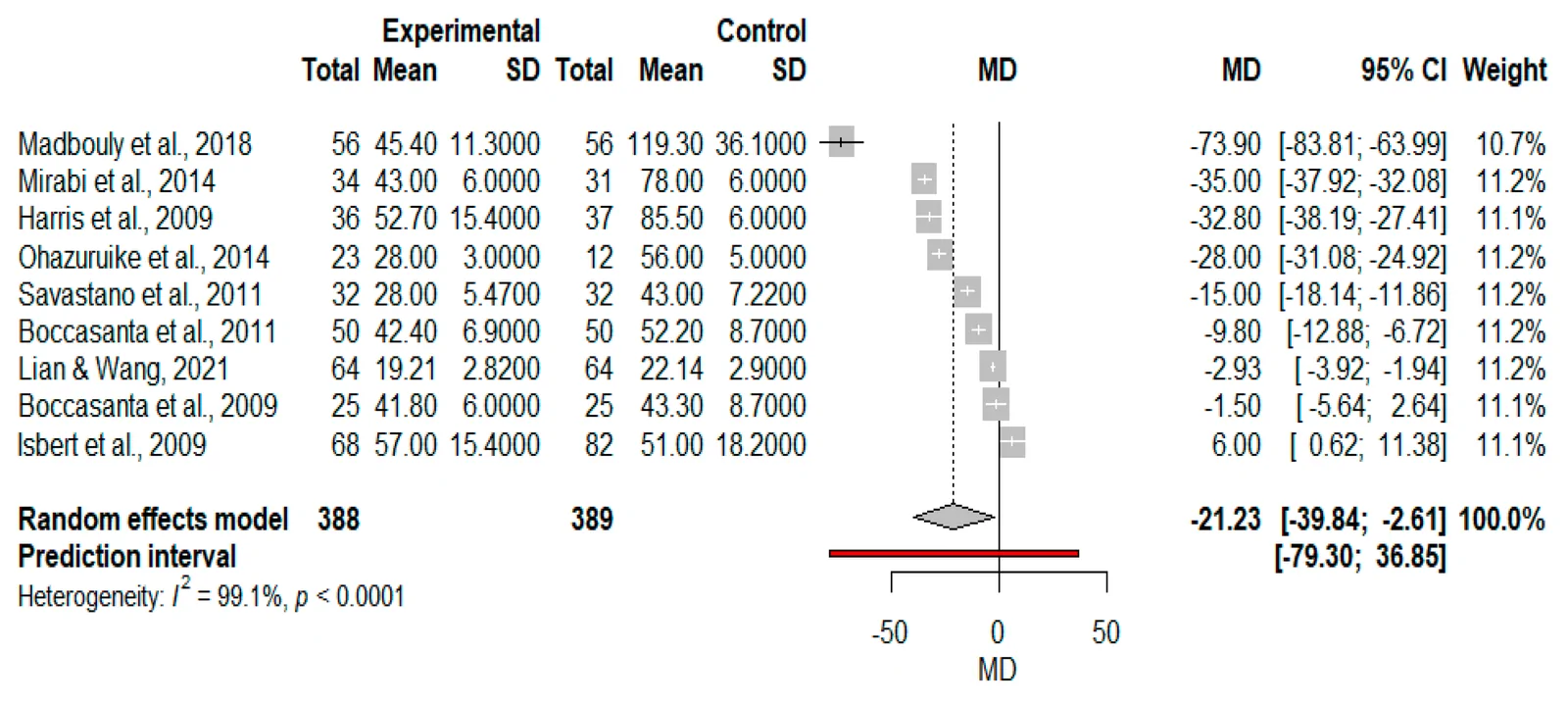
Shows a forest plot of the pooled MD of operation time in minutes comparing STARR surgery with control management in ODS patients [11,14,15,19,20,23,24,25,26].

**Figure 7:**
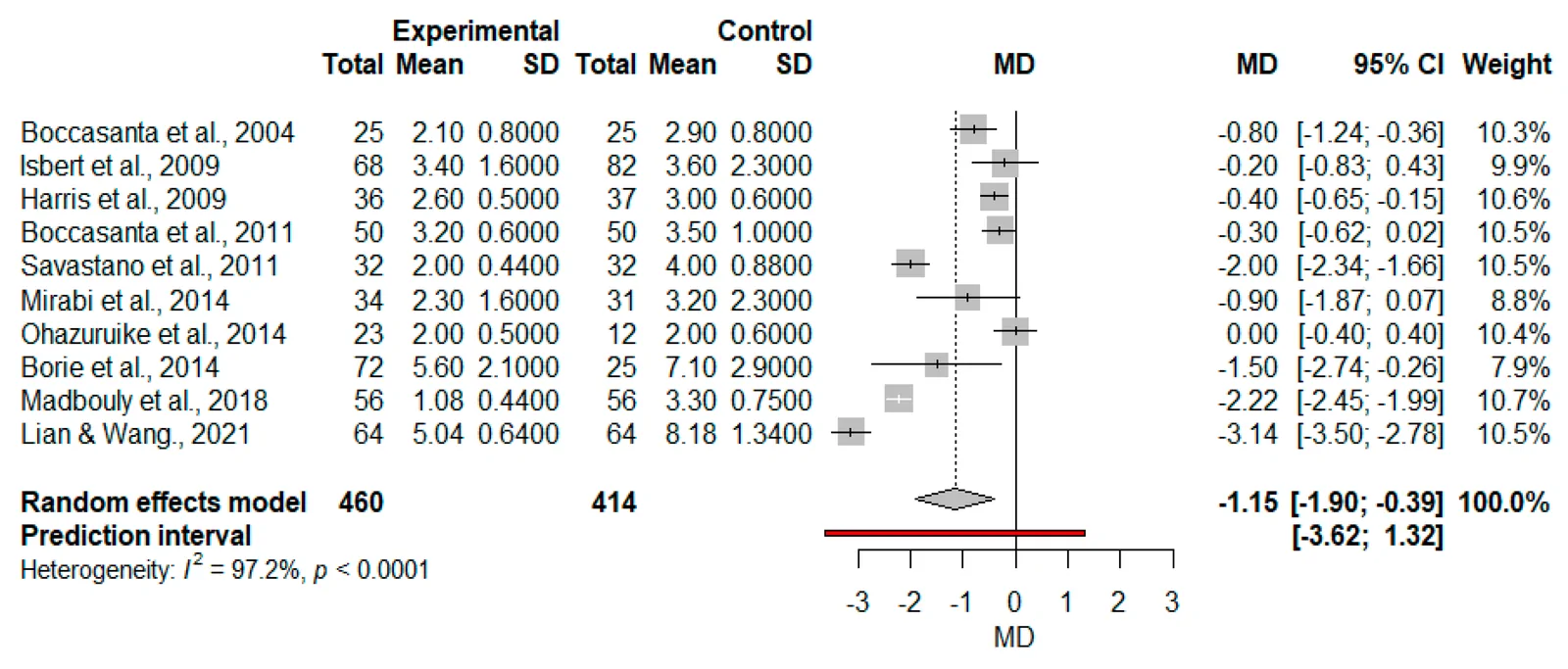
Forest plot of the pooled MD of LOS in days comparing STARR surgery with control management in ODS patients [11,14,15,19,20,21,23,24,25,26].

**Table 1:** Characteristics of the included studies.

Author, Year, Country	Study Design	Sample Size, Female %	Age (STARR, Control)	Cause of ODS	STARR Device	Diagnostic Criteria	Population Criteria	Control Name	Follow Up Period	ROB2	NOS
Biviano et al., 2011, Italy [18]	Observational comparative study	60, 96%	51.0, 53.0	Rectocele or rectal intussusception	Not mentioned	Anorectal manometry and defecography	No prior anorectal surgery, no abnormalities, or systemic disease Radiological evidence, No prior treatment with oral macrogol or laxatives Not pregnant	Conservative treatment (macrogol)	41	NA	Fair
Isbert et al., 2010, Germany [14]	Cohort study	150, 100%	65.0, 66.0	Rectocele and/or rectal intussusception	PPH-STARR	symptoms, clinical examination, anal manometry and video-proctoscopy & dynamic defecography	No malignancy	Contour-Transtar	12	NA	Fair
Mirabi et al., 2014, Iran [24]	Retrospective comparative	81, 100%	49.5, 47.5	Intussusception or prolapse	Not mentioned	anoscopy, colonoscopy, defecography, colon transit time, endoanal sonography, and anal manometry	Female patients with ODS	Delorme operation	12	NA	Fair
Madbouly et al., 2019, Egypt [26]	RCT	112, 74%	76.7, 73.0	Rectocele and rectal intussusception	Contour Transtar	Fluoroscopic defecography	Pelvic structural abnormalities, not response to medication	LVR	12	High risk	NA
Boccasanta et al., 2004, Italy [11]	RCT	50, 100%	54.6, 53.2	Descending perineum, rectocele, and rectal intussusception	PPH-01	Fluoroscopic defecography	≥3 ODS, symptoms >6 months on conservative therapy, rectocele ≥3 cm + intussusception ≥10 mm, no (genital prolapse/cystocele requiring surgery, fecal incontinence, sphincter defects >20%, recurrent rectocele/enterocele, mental disorders, megarectum >6.5 cm)	STAPL	23	High risk	NA
Ohazuruike et al., 2014, Italy [20]	cohort study	35, 82%	53.0, 58.8	Internal rectal intussusception and rectocele	PPH-01	clinical evaluation, proctoscopy, defecography, anorectal manometry, and endoanal ultrasonography.	failure of 6 months of medical therapy, ≥3 symptoms, defecography-confirmed structural abnormality, and ODS score ≥12. Exclusion: non-relaxing puborectalis, genital prolapse requiring surgery, fecal incontinence, mental disorders, sphincter injuries, pelvic floor dyssynergia.	Delorme operation	15	NA	Fair
Boccasanta et al., 2011, Italy [19]	RCT	100, 100%	54.8, 57.1	Rectal intussusception/internal rectal prolapse and rectocele	PPH-01	Clinical symptoms, defecography, colpocystodefecography	No prior rectal surgery, IRP grade I, rectocele <3 cm, ODS <15, continence >3, low anal pressures, concomitant enterocele/cystocele/genital prolapse, psychiatric disease, surgical contraindications	TRANSTAR	36.3	High risk	NA
Lian & Wang, 2021, China [25]	Retrospective comparative	124, 73%	52.93, 53.0	rectal prolapse, rectocele, perineal descent, pelvic floor spasm	Not mentioned	Clinical symptoms + defecography findings (implied); Rome criteria	Meets OOC diagnostic criteria; difficult/incomplete defecation; senses of obstruction/prolapse; confirmed anatomical abnormalities. There is no severe organ disease, cancer, coagulopathy, hematologic or infectious disease, contraindication to anesthesia, or cognitive or neurological disease.	Bresler operation	36	NA	Good
Harris et al., 2009, USA [23]	Retrospective cohort	73, 100%	53.2, 58.0	Rectocele and/or rectal intussusception	PPH01, PPH03	Defecography;	Inclusion: ODS symptoms, confirmed defecography. Exclusion: Incontinence to solid stool, full-thickness prolapse, fistula, infection, IBD, cancer, severe pelvic floor dysfunction, psychiatric impairment	TVR	7.8	NA	Fair
Lehur et al., 2008, France [22]	RCT	119, 100%	56.0, 56.0	Rectocele ± rectal intussusception	PPH01	Dynamic defecography, ODS score	Inclusion: female, age ≥ 18, ODS score ≥ 7, confirmed defecography. Exclusion: external sphincter injury, fecal incontinence, enterocele requiring surgery, colpocele/cystocele needing combined repair	Biofeedback	15	High risk	NA
Savastano et al., 2011, Italy [15]	Observational comparative study	64, 100%	54.1, 60.5	Rectocele and internal rectal prolapse	PPH01	Symptoms + defecography confirmation; ODS score	Not mentioned	Contour-Transtar	27	NA	Good
Borie et al., 2014, France [21]	Observational comparative study	52, 100%	56.0, 60.0	Rectocele ± recto-anal intussusception causing outlet obstruction	Not mentioned	ODS score, defecography (rectocele > 3 cm), anorectal manometry, endoanal ultrasonography, proctoscopy ± colonoscopy	Dyschezia with rectocele ± intussusception Outlet obstruction >6 months Failed medical treatment (biofeedback/laxatives)	LVR	18	NA	Fair

LVR: Laparoscopic Ventral Rectopexy; NA: Not applied; STAPL: Single Stapled Trans-Anal Prolapsectomy with Perineal Levatorplasty; TRANSTAR: Stapled transanal rectal resection with curved multifare stapler; TPS: Transperineal Sonography; TVR: Transvaginal rectocele repair.

## Data Availability

Data will be available with the corresponding author to be presented on reasonable request.

## Supplementary Materials

The following supporting information can be downloaded at: https://www.mdpi.com/article/10.3390/jcm15197347/s1.

## Abbreviations

The following abbreviations are used in this manuscript:

STARR	Stapled Transanal Rectal Resection
ODS	Obstructed Defecation Syndrome
RCT	Randomized Controlled Trial
SMD	Standardized Mean Difference
OR	Odds Ratio
MD	Mean Difference
CI	Confidence Interval
I^2^	Higgins’ Inconsistency Statistic (Heterogeneity)
PRISMA	Preferred Reporting Items for Systematic Reviews and Meta-Analyses
PROSPERO	International Prospective Register of Systematic Reviews
LVMR/LVR	Laparoscopic Ventral Mesh Rectopexy
STAPL	Single Stapled Trans-Anal Prolapsectomy with Perineal Levatorplasty
TPS	Transperineal Sonography (used as combined STARR+TPS technique)
TVR	Transvaginal Rectocele Repair
CCCS	Cleveland Clinic Constipation Score
LOS	Length of Stay
ROB2	Revised Cochrane Risk-of-Bias Tool (for RCTs)
NOS	Newcastle–Ottawa Scale
PPH-01	Procedure for Prolapse and Hemorrhoids (circular stapler device)
MEDLINE	Medical Literature Analysis and Retrieval System Online
CENTRAL	Cochrane Central Register of Controlled Trials
WOS	Web of Science
CT	Computed Tomography
ODS Score	Obstructed Defecation Syndrome Score (Altomare scoring instrument)

## References

[1] Gray J.R. (2011). What is chronic constipation? Definition and diagnosis. Can. J. Gastroenterol. Hepatol..

[2] Lacy B.E., Mearin F., Chang L., Chey W.D., Lembo A.J., Simren M., Spiller R. (2016). Bowel disorders. Gastroenterology.

[3] Camilleri M., Ford A.C., Mawe G.M., Dinning P.G., Rao S.S., Chey W.D., Simrén M., Lembo A., Young-Fadok T.M., Chang L. (2017). Chronic constipation. Nat. Rev. Dis. Primers.

[4] Lee Y.Y., Kamani L., Piscoya A., Dumitrascu D., Simadibrata D.M., Makharia G.K., Espinoza-Ríos J., Remes-Troche J.M., Setshedi M., Rahman M.M. (2025). A Global Cascade Approach to Diagnosis and Management of Chronic Constipation.

[5] Liu W.C., Wan S.L., Yaseen S.M., Ren X.H., Tian C.P., Ding Z., Zheng K.Y., Wu Y.H., Jiang C.Q., Qian Q. (2016). Transanal surgery for obstructed defecation syndrome: Literature review and a single-center experience. World J. Gastroenterol..

[6] Racaniello E., Terzoni S., Accardi R., Ricci C., Boccasanta P., Destrebecq A. (2015). Quality of life of patients undergoing surgery for obstructed defecation syndrome: A before-after study. Int. J. Surg..

[7] Ripamonti L., Guttadauro A., Lo Bianco G., Rennis M., Maternini M., Cioffi G., Chiarelli M., De Simone M., Cioffi U., Gabrielli F. (2022). Stapled Transanal Rectal Resection (Starr) in the Treatment of Obstructed Defecation: A Systematic Review. Front. Surg..

[8] Andromanakos N., Skandalakis P., Troupis T., Filippou D. (2006). Constipation of anorectal outlet obstruction: Pathophysiology, evaluation and management. J. Gastroenterol. Hepatol..

[9] Perek A., Ergün S. (2021). Obstructed defecation syndrome. Turk. J. Colorectal Dis..

[10] Altomare D.F., Spazzafumo L., Rinaldi M., Dodi G., Ghiselli R., Piloni V. (2008). Set-up and statistical validation of a new scoring system for obstructed defaecation syndrome. Colorectal Dis..

[11] Boccasanta P., Venturi M., Salamina G., Cesana B.M., Bernasconi F., Roviaro G. (2004). New trends in the surgical treatment of outlet obstruction: Clinical and functional results of two novel transanal stapled techniques from a randomised controlled trial. Int. J. Colorectal Dis..

[12] Heymen S., Scarlett Y., Jones K., Ringel Y., Drossman D., Whitehead W.E. (2007). Randomized, controlled trial shows biofeedback to be superior to alternative treatments for patients with pelvic floor dyssynergia-type constipation. Dis. Colon Rectum.

[13] Corman M.L., Carriero A., Hager T., Herold A., Jayne D.G., Lehur P.A., Lomanto D., Longo A., Mellgren A.F., Nicholls J. (2006). Consensus conference on the stapled transanal rectal resection (STARR) for disordered defaecation. Colorectal Dis..

[14] Isbert C., Reibetanz J., Jayne D.G., Kim M., Germer C.T., Boenicke L. (2010). Comparative study of contour transtar and STARR procedure for the treatment of obstructed defecation syndrome (ODS)—Feasibility, morbidity and early functional results. Colorectal Dis..

[15] Savastano S., Valenti G., Cavallin F., Missaglia C. (2011). STARR With PPH-01 and CCS30 Contour Transtar for Obstructed Defecation Syndrome. Surg. Innov..

[16] Deeks J.J., Bossuyt P.M., Leeflang M.M., Takwoingi Y. (2023). Cochrane Handbook for Systematic Reviews of Diagnostic Test Accuracy.

[17] Gualdi-Russo E., Zaccagni L. (2026). The Newcastle–Ottawa Scale for assessing the quality of studies in systematic reviews. Publications.

[18] Biviano I., Badiali D., Candeloro L., Habib F.I., Mongardini M., Caviglia A., Anzini F., Corazziari E.S. (2011). Comparative outcome of stapled trans-anal rectal resection and macrogol in the treatment of defecation disorders. World J. Gastroenterol..

[19] Boccasanta P., Venturi M., Roviaro G. (2011). What is the benefit of a new stapler device in the surgical treatment of obstructed defecation? Three-year outcomes from a randomized controlled trial. Dis. Colon Rectum.

[20] Ohazuruike N.L., Martellucci J., Menconi C., Panicucci S., Toniolo G., Naldini G. (2014). Short-term results after STARR versus internal Delorme for obstructed defecation: A non-randomized prospective study. Updates Surg..

[21] Borie F., Bigourdan J.-M., Pissas M.-H., Guillon F. (2014). Laparoscopic ventral rectopexy for the treatment of outlet obstruction associated with recto-anal intussusception and rectocele: A valid alternative to STARR procedure in patients with anal sphincter weakness. Clin. Res. Hepatol. Gastroenterol..

[22] Lehur P.A., Stuto A., Fantoli M., Villani R.D., Queralto M., Lazorthes F., Hershman M., Carriero A., Pigot F., Meurette G. (2008). Outcomes of Stapled Transanal Rectal Resection vs. Biofeedback for the Treatment of Outlet Obstruction Associated with Rectal Intussusception and Rectocele: A Multicenter, Randomized, Controlled Trial. Dis. Colon Rectum.

[23] Harris M.A., Ferrara A., Gallagher J., DeJesus S., Williamson P., Larach S. (2009). Stapled Transanal Rectal Resection vs. Transvaginal Rectocele Repair for Treatment of Obstructive Defecation Syndrome. Dis. Colon Rectum.

[24] Mirabi N., Fazlani M., Raisee R. (2014). Comparing the Outcomes of Stapled Transanal Rectal Resection, Delorme Operation and Electrotherapy Methods Used for the Treatment of Obstructive Defecation Syndrome. Iran. J. Med. Sci..

[25] Lian T., Wang N. (2021). Stapled trans-anal rectal resection can improve constipation symptoms and inflammatory reaction of patients with outlet obstructive constipation. Am. J. Transl. Res..

[26] Madbouly K.M., Mohii A.D. (2019). Laparoscopic ventral rectopexy versus stapled transanal rectal resection for treatment of obstructed defecation in the elderly: Long-term results of a prospective randomized study. Dis. Colon Rectum.

[27] Mandovra P., Kalikar V., Patankar R.V. (2020). Clinical outcomes of stapled transanal rectal resection (STARR) for obstructed defecation syndrome: Is STARR really a star on long-term follow-up?. World J. Colorectal Surg..

[28] Andreoli R., Zampieri N., Orlandi S., Geccherle A., Castellani R.L. (2024). Long time consequences after STARR procedure: Report of 15 years experience and medico-legal perspective. Am. J. Surg..

[29] Renzi A., Marano L., Talento P., Brusciano L., Pezzolla A., Izzo D., Antropoli C., D’Aniello F., Di Sarno G., Minieri G. (2025). Transverse perineal support improves long-term outcomes in patients undergoing stapled transanal rectal resection for obstructed defecation syndrome: A multicenter observational case-control study. Ann. Coloproctology.

[30] Mohamed O.R., Shafik A.A., Elbarmelgi M.Y., Basiouny H.M.A., Hassan A.A., Mostafa M.T.M. (2025). Stapled Trans-anal Rectal Resection Using Single Versus Double Stapler in Surgical Management of Obstructed Defecation: A Randomised Clinical Trial. Indian J. Surg..

[31] Mascagni D., Panarese A., Eberspacher C., Grimaldi G., Pontone S., Sorrenti S., Pironi D. (2020). Standard stapled transanal rectal resection versus stapled transanal rectal resection with one high-volume stapler to prevent complications in the elderly. Minerva Chir..

[32] Menconi C., Fabiani B., Giani I., Martellucci J., Toniolo G., Naldini G. (2016). Persistent anal and pelvic floor pain after PPH and STARR: Surgical management of the fixed scar staple line. Int. J. Colorectal Dis..

[33] Chen X., Chu Q., Peng Y., Chen Y., Kaye A.D., Liu H., Yang J., Wang T., Yu W. (2025). Clinical practice guidelines for postoperative pain management in adults (2024 edition). J. Anesth. Transl. Med..

[34] Guttadauro A., Chiarelli M., Maternini M., Baini M., Pecora N., Gabrielli F. (2018). Value and limits of stapled transanal rectal repair for obstructed defecation syndrome: 10 years-experience with 450 cases. Asian J. Surg..

[35] Hasan H.M., Hasan H.M. (2012). Stapled transanal rectal resection for the surgical treatment of obstructed defecation syndrome associated with rectocele and rectal intussusception. Int. Sch. Res. Not..

[36] Abdwahed M., Elbarbary M.G., Ibrahim A.-A.H. (2019). STARR operation in management of patients with rectal intussusception: Short term results. Ain Shams J. Surg..

[37] Marra A.A., Varrella C., Caruso C., Simonelli I., Parello A., Litta F., Campennì P., Pagano M., Ratto C. (2026). Focused analysis of the surgical management for recurrence of internal rectal prolapse and obstructed defecation syndrome after stapled rectal resection procedures: A long-term single-center experience. Int. J. Colorectal Dis..

[38] Morsy A.M., Elkhateeb A., Nashed G.A., Makhlouf G.A., Hanna R.S. (2022). Laparoscopic or transanal approach: Which is better for the management of obstructed defecation?. Egypt. J. Surg..

[39] Tsunoda A., Yasuda N., Yokoyama N., Kamiyama G., Kusano M. (2003). Delorme’s procedure for rectal prolapse: Clinical and physiological analysis. Dis. Colon Rectum.

[40] Farouk R., Bhardwaj R., Phillips R., Dehn T. (2009). Stapled transanal resection of the rectum (STARR) for the obstructed defaecation syndrome. Ann. R. Coll. Surg. Engl..

[41] Chung A.S., Ballatori A., Ortega B., Min E., Formanek B., Liu J., Hsieh P., Hah R., Wang J.C., Buser Z. (2021). Is Less Really More? Economic Evaluation of Minimally Invasive Surgery. Glob. Spine J..

