# Supplementary material for: Efficacy and Safety of Stapled Transanal Rectal Resection (STARR) for Obstructed Defecation Syndrome: A Systematic Review and Meta-Analysis of Comparative Studies
Source: J Clin Med. 2026 Sep 22;15(19):7347. doi: 10.3390/jcm15197347 (PMC13666251; doi:10.3390/jcm15197347)
Supplement: Supplementary file 1 [file jcm-15-07347-s001.zip › jcm-4429821-supplementary.pdf]

Supplementary File S1

PubMed<br>159<br>articles	(("stapled transanal rectal resection"[Title/Abstract] OR "STARR procedure"[Title/Abstract] OR "STARR surgery"[Title/Abstract] OR "stapled trans-anal rectal resection"[Title/Abstract] OR "transanal stapled rectal resection"[Title/Abstract] OR "laparoscopic ventral mesh rectopexy"[Title/Abstract] OR "ventral mesh rectopexy"[Title/Abstract] OR "laparoscopic ventral rectopexy"[Title/Abstract] OR "ventral rectopexy"[Title/Abstract] OR "internal delorme"[Title/Abstract] OR "internal delorme's procedure"[Title/Abstract] OR "internal delorme procedure"[Title/Abstract] OR "mucosal proctoplasty"[Title/Abstract] OR "transanal mucosal resection"[Title/Abstract] OR "contour transtar"[Title/Abstract] OR "transtar procedure"[Title/Abstract] OR "contour transtar procedure"[Title/Abstract]) AND ("Anal Canal"[MeSH Terms] OR "Anal Canal"[Title/Abstract] OR Biofeedback[MeSH Terms] OR Biofeedback[Title/Abstract] OR Psychology[MeSH Terms] OR Psychology[Title/Abstract] OR Constipation[MeSH Terms] OR Constipation[Title/Abstract] OR Defecation[MeSH Terms] OR Defecation[Title/Abstract] OR Rectum[MeSH Terms] OR Rectum[Title/Abstract] OR "Intestinal Obstruction"[MeSH Terms] OR "Intestinal Obstruction"[Title/Abstract] OR "Rectal Diseases"[MeSH Terms] OR "Rectal Diseases"[Title/Abstract] OR Hypertrophy[MeSH Terms] OR Hypertrophy[Title/Abstract] OR "Rectal Prolapse"[MeSH Terms] OR "Rectal Prolapse"[Title/Abstract] OR Intussusception[MeSH Terms] OR Intussusception[Title/Abstract])) AND ("Postoperative Complications"[MeSH Terms] OR "Postoperative Complication*"[Title/Abstract] OR "Complication*, Postoperative"[Title/Abstract] OR "Postoperative Complication"[Title/Abstract] OR "Postoperative Complications"[Title/Abstract])	13-1-2026<br>Done<br>by………
WOS<br>487	TS=(("stapled transanal rectal resection" OR "STARR procedure" OR "STARR surgery" OR "stapled trans-anal rectal resection" OR "transanal stapled rectal resection" OR "laparoscopic ventral mesh rectopexy" OR "ventral mesh rectopexy" OR "laparoscopic ventral rectopexy" OR "ventral rectopexy" OR "internal delorme" OR "mucosal proctoplasty" OR "transanal mucosal resection" OR "contour transtar" OR "transtar procedure") AND (anal canal OR biofeedback OR psycholog* OR constipat* OR defecat* OR rectum OR rectal OR "intestinal obstruction" OR "rectal prolapse" OR intussusception OR "obstructed defecation" OR "anismus" OR "dyssynergic defecation") AND (complicat* OR "postoperative complicat*" OR "adverse event*" OR morbidity OR "functional outcome*" OR "quality of life"))	13-1-2026<br>Done by<br>…….
Sage<br>59	("stapled transanal rectal resection" OR "STARR procedure" OR "STARR surgery" OR "transanal stapled rectal resection" OR "ventral rectopexy" OR "internal delorme" OR "mucosal proctoplasty" OR "transtar") AND (rectal prolapse OR "obstructed defecation" OR constipat* OR defecat* OR intussusception OR anismus) AND	13-1-2026<br>Done by…..

	(complicat* OR "postoperative" OR morbidity OR "functional outcome*")	
Cochrane 102	(("stapled transanal rectal resection" OR "STARR procedure" OR "STARR surgery" OR "stapled trans-anal rectal resection" OR "transanal stapled rectal resection" OR "laparoscopic ventral mesh rectopexy" OR "ventral mesh rectopexy" OR "laparoscopic ventral rectopexy" OR "ventral rectopexy" OR "internal delorme" OR "internal delorme's procedure" OR "internal delorme procedure" OR "mucosal proctoplasty" OR "transanal mucosal resection" OR "contour transtar" OR "transtar procedure" OR "contour transtar procedure") AND (MeSH descriptor: [Anal Canal] explode all trees OR MeSH descriptor: [Biofeedback] explode all trees OR MeSH descriptor: [Psychology] explode all trees OR MeSH descriptor: [Constipation] explode all trees OR MeSH descriptor: [Defecation] explode all trees OR MeSH descriptor: [Rectum] explode all trees OR MeSH descriptor: [Intestinal Obstruction] explode all trees OR MeSH descriptor: [Rectal Diseases] explode all trees OR MeSH descriptor: [Hypertrophy] explode all trees OR MeSH descriptor: [Rectal Prolapse] explode all trees OR MeSH descriptor: [Intussusception] explode all trees OR "Anal Canal" OR Biofeedback OR Psychology OR Constipation OR Defecation OR<br>Rectum OR "Intestinal Obstruction" OR "Rectal Diseases" OR Hypertrophy OR "Rectal Prolapse" OR Intussusception) AND (MeSH descriptor: [Postoperative Complications] explode all trees OR "Postoperative Complication*" OR "Complication*, postoperative" OR "Postoperative Complications"))	15-1-2026 Done by……
ProQuest 15 article	("stapled transanal rectal resection" OR "STARR procedure" OR "STARR surgery" OR "stapled trans-anal rectal resection" OR "transanal stapled rectal resection" OR "laparoscopic ventral mesh rectopexy" OR "ventral mesh rectopexy" OR "laparoscopic ventral rectopexy" OR "ventral rectopexy" OR "internal delorme" OR "internal delorme's procedure" OR "internal delorme procedure" OR "mucosal proctoplasty" OR "transanal mucosal resection" OR "contour transtar" OR "transtar procedure" OR "contour transtar procedure")<br>AND<br>("Anal Canal" OR Biofeedback OR Psychology OR Constipation OR Defecation OR Rectum OR "Intestinal Obstruction" OR "Rectal Diseases" OR Hypertrophy OR "Rectal Prolapse" OR intussusception)<br>AND<br>("Postoperative Complication*" OR "Postoperative Complications" OR "Complication*, Postoperative")	15-1-2026 Done by ……

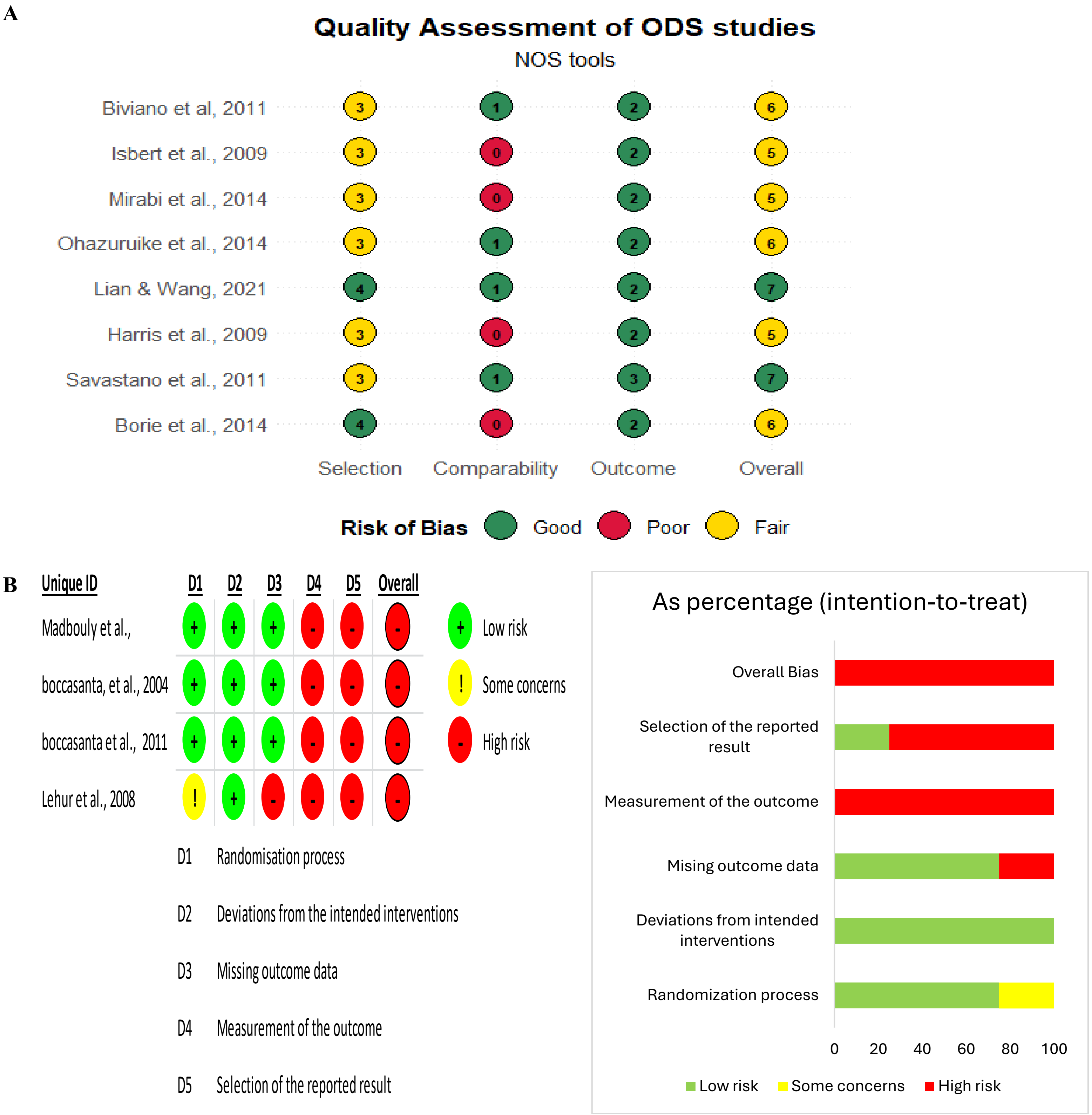

Figure S1: Quality assessment of the included studies in the meta-analysis (A) New-Castle Ottawa (NOS) tool assessment. (B) ROB2 tool assessment

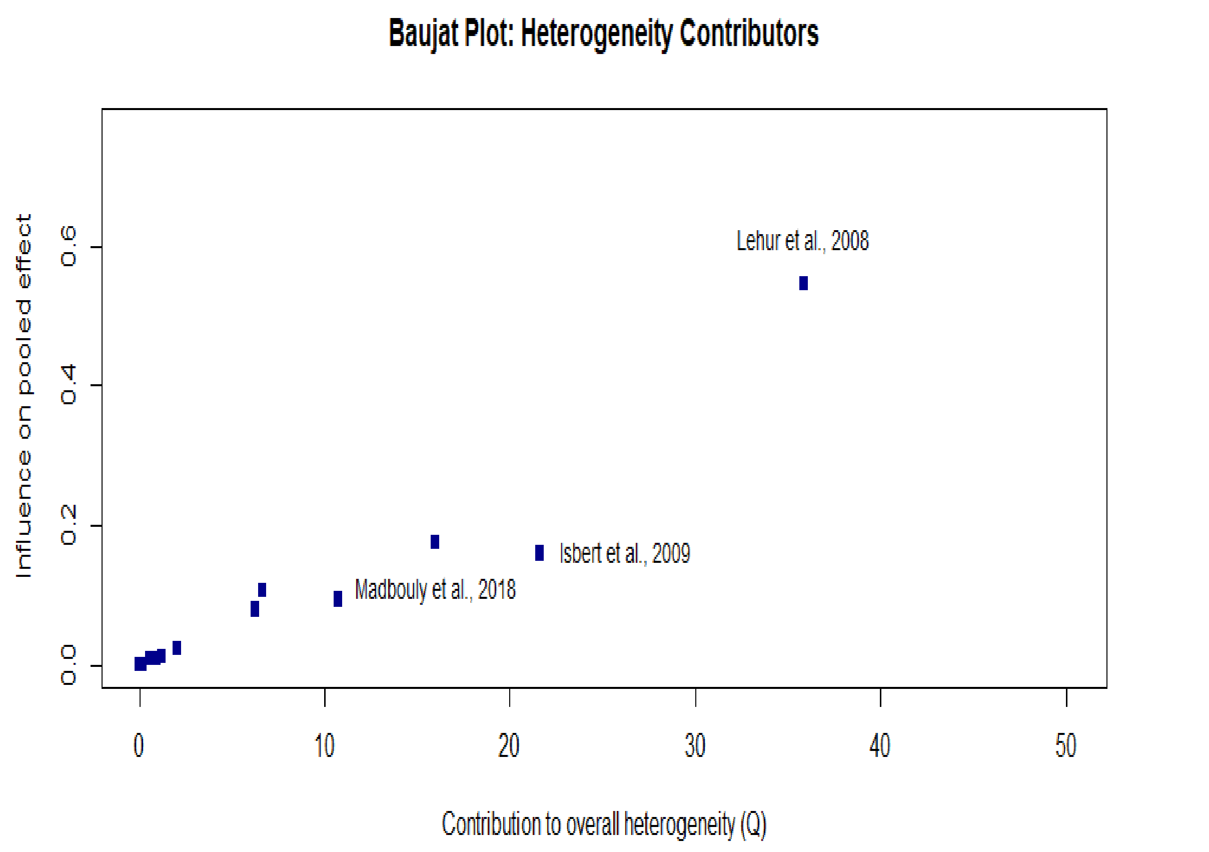

Figure S2A: Baujat plot for ODS conistipation score checking for influential and outliers,

showing contribution of each included study on the pooled effect and the

overall heterogeneity

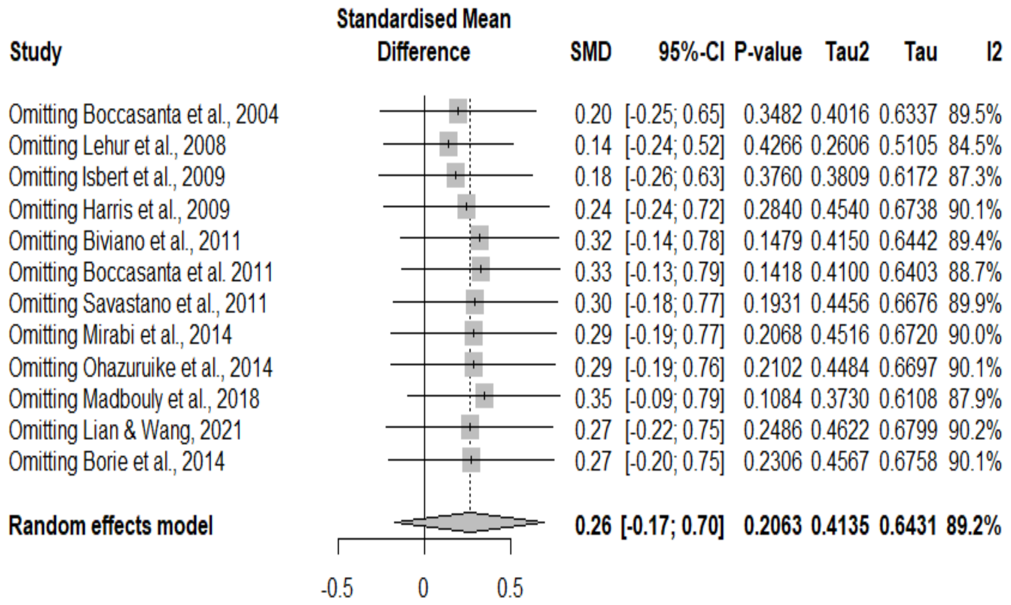

Figure S2B: Pooled effect (SMD, CI) and heterogeneity I^2 of ODS conistipation score after conducting leave one sensitivity analysis per study.

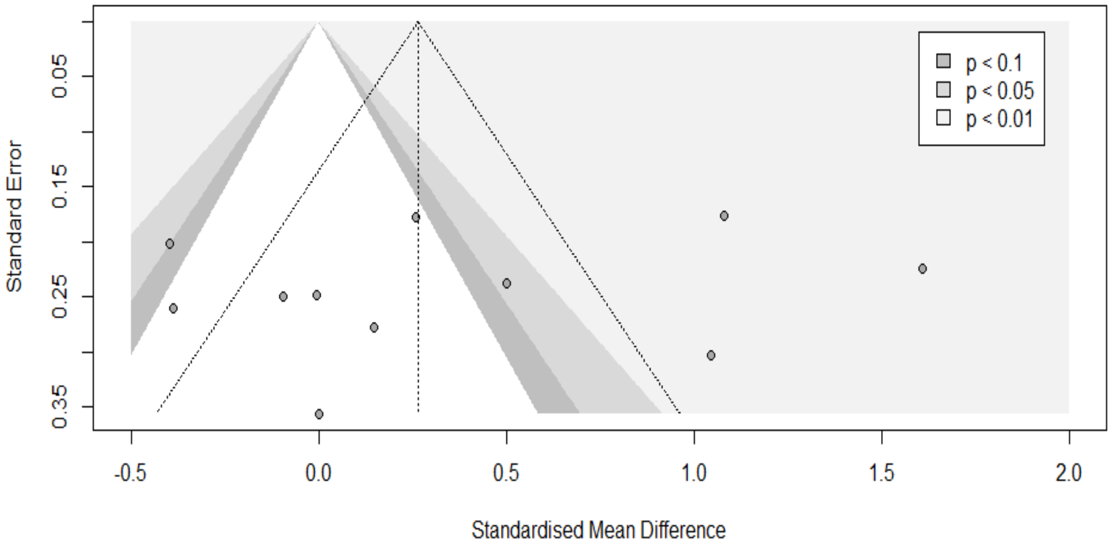

Figure S2C: Funnel plot showing publication bias for published studies regarding SMD of ODS constipation score

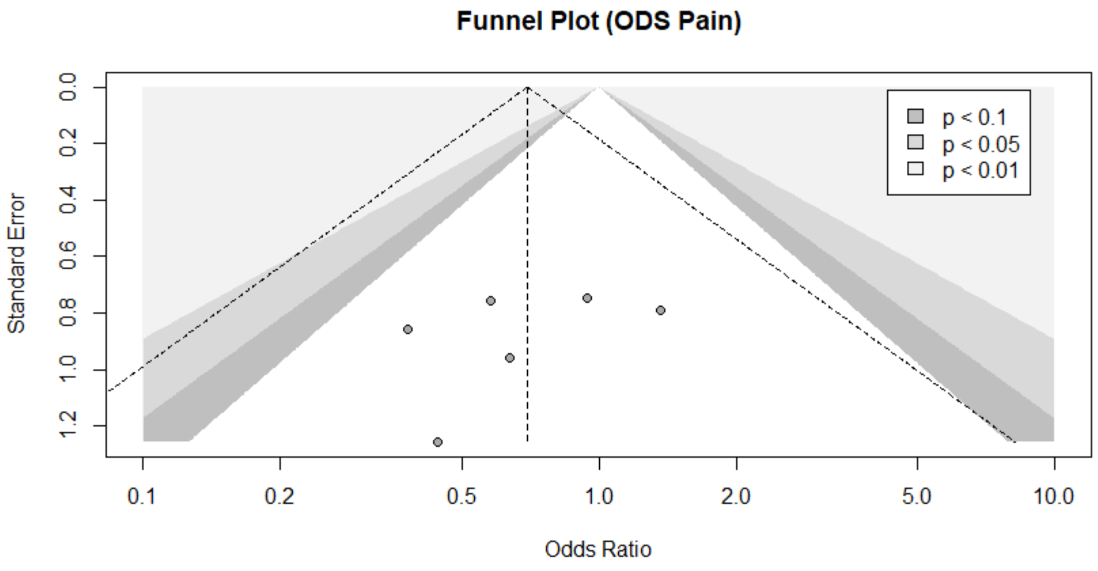

Figure S2D: Funnel plot of publication bias for published studies regarding OR of pain

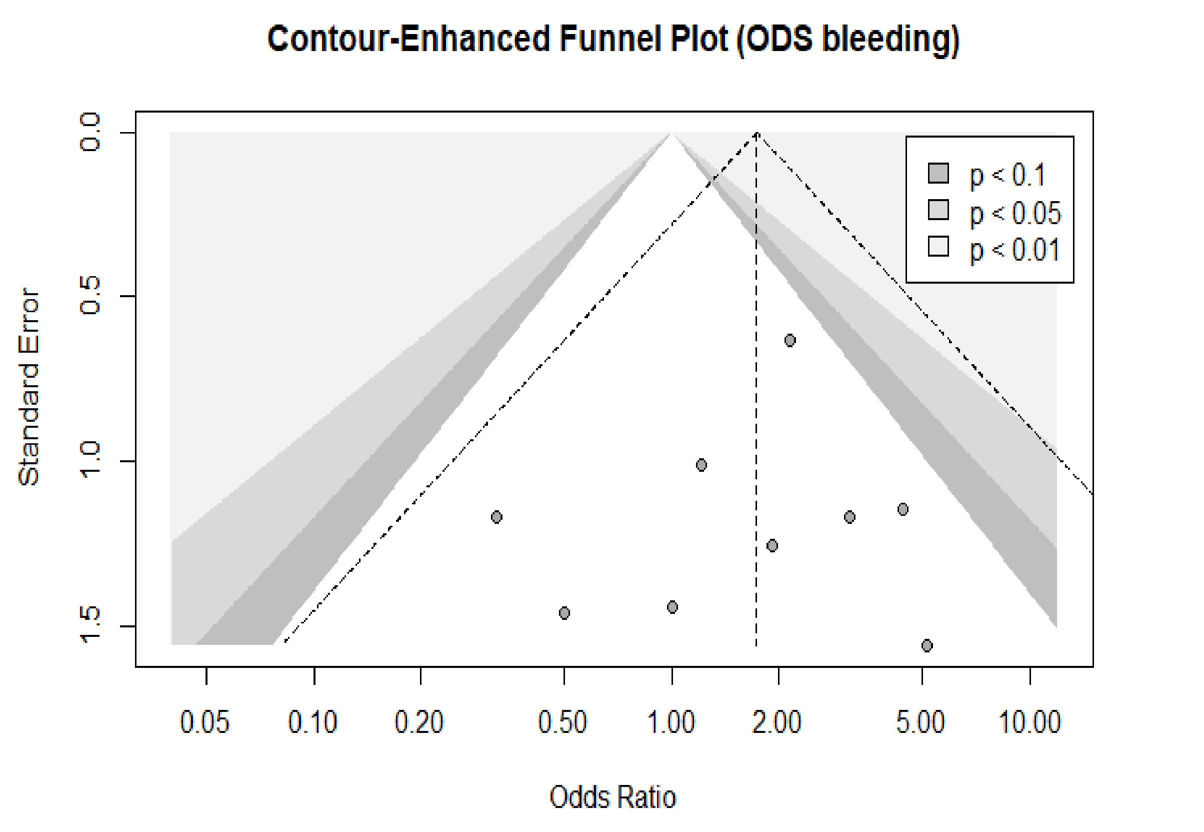

Figure S2E: Funnel plot of publication bias for published studies regarding OR of bleeding

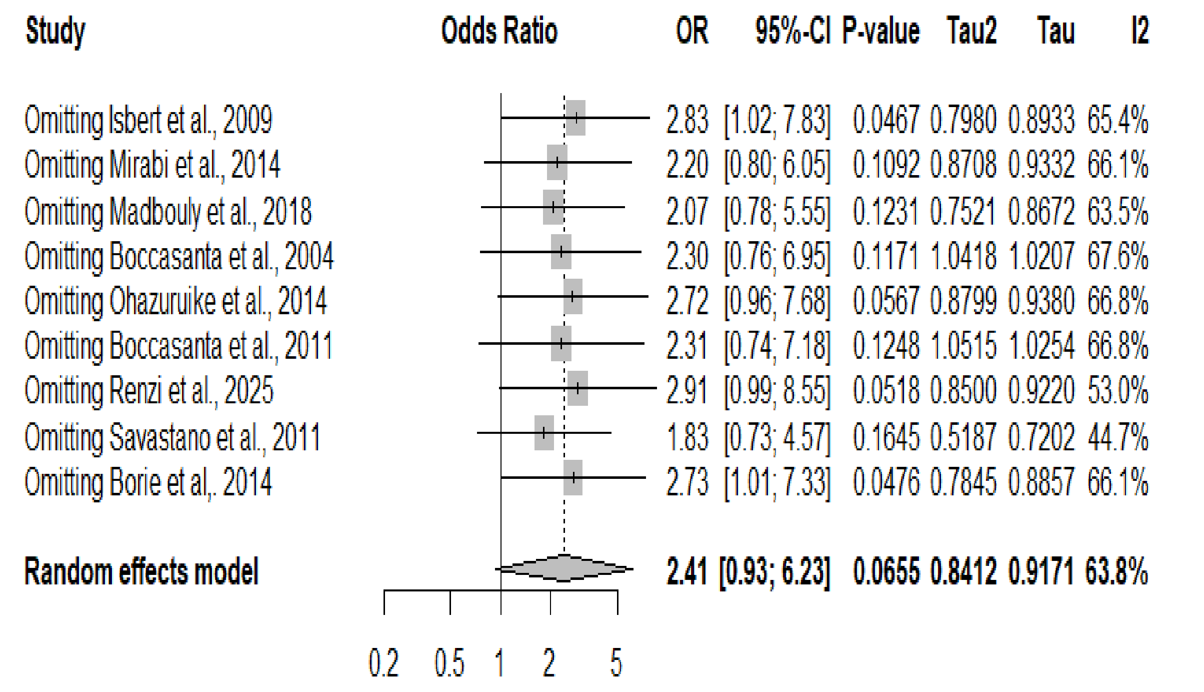

Figure S2F: Pooled effect (OR, CI) and heterogeneity I² of postoperative urge /incontinence after conducting leave one sensitivity analysis per study

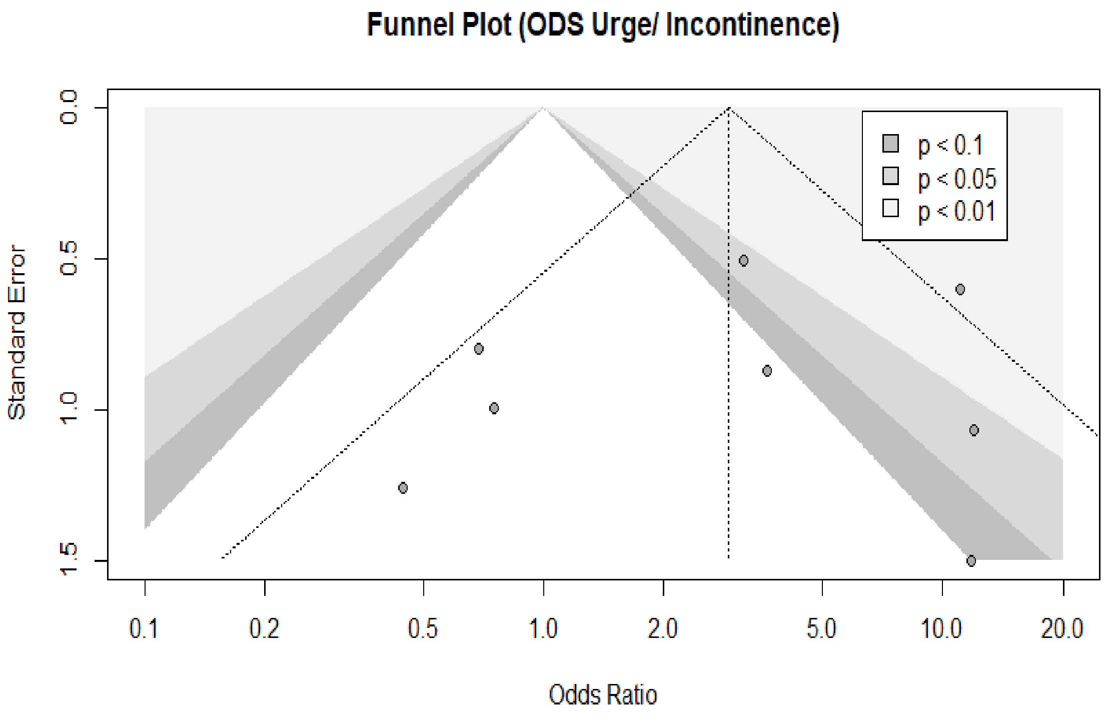

Figure S2G: Funnel plot of publication bias for published studies regarding OR of urge/ incontinence

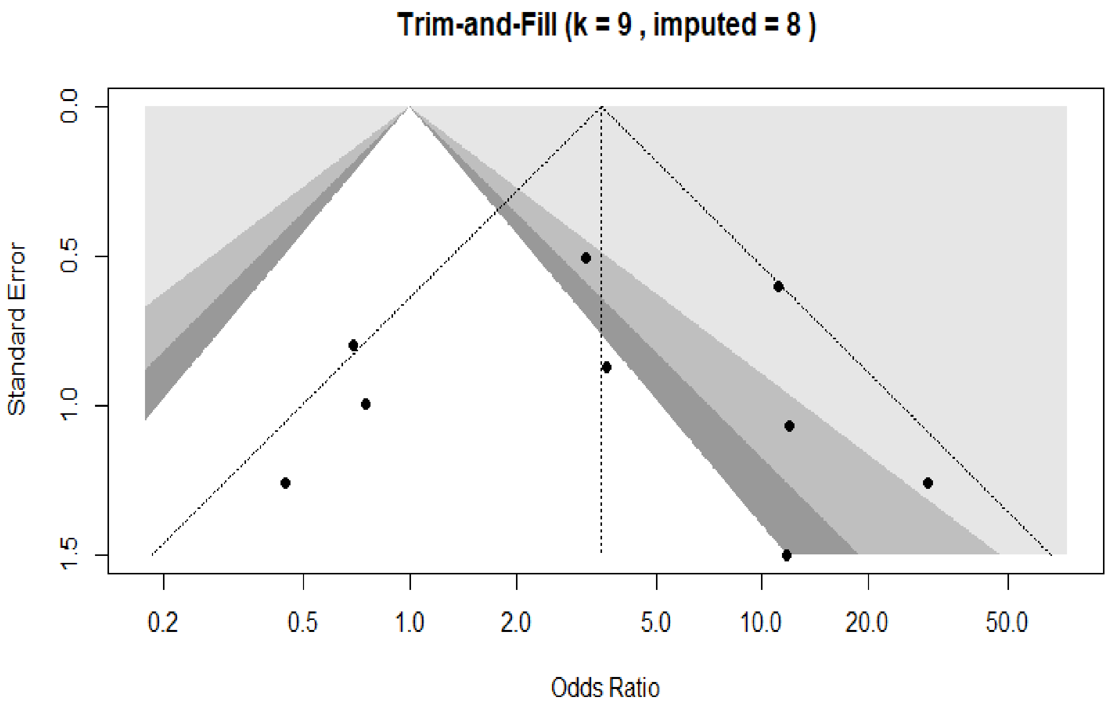

Figure S2H: Trimfill plot of postoperative pooled OR of urge/ incontinence

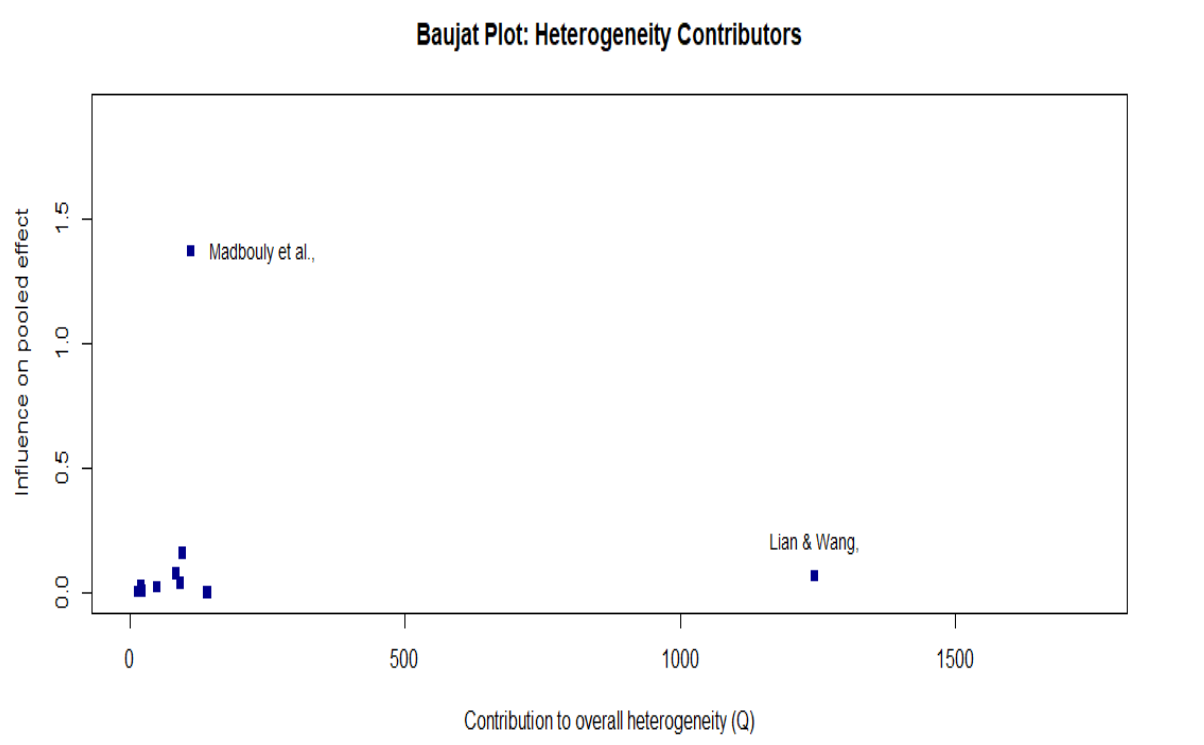

Figure S2I: Baujat plot for <u>operation time</u> checking for influential and outliers, showing contribution of each included study on the pooled effect and the overall heterogeneity

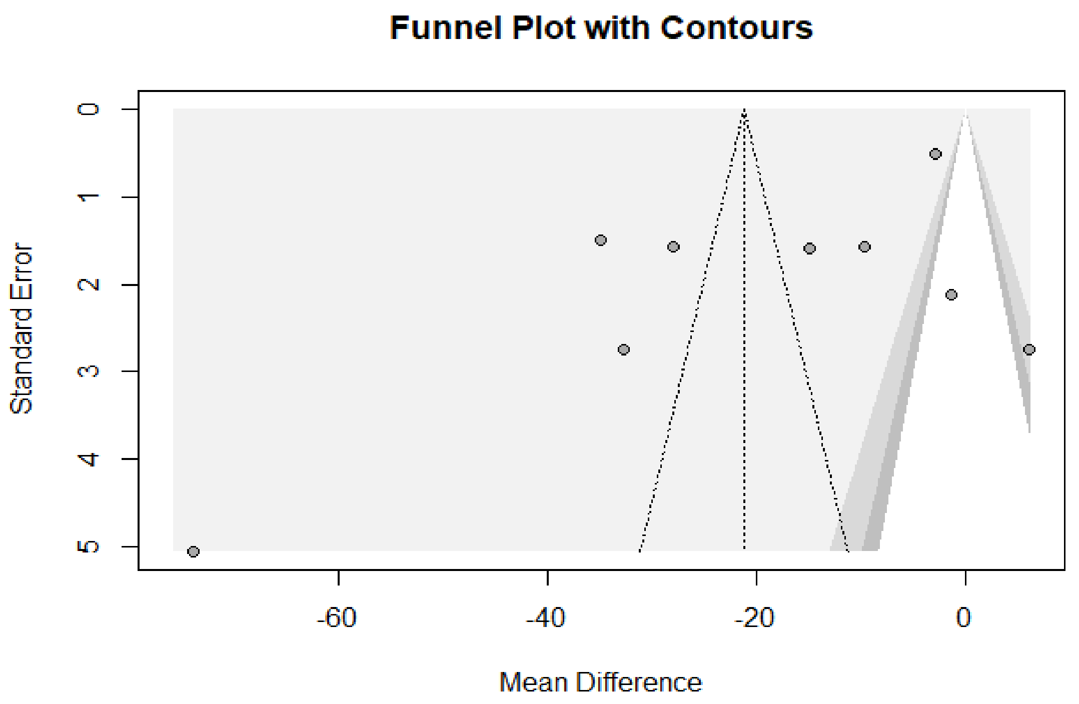

Figure S2J: Funnel plot of publication bias for published studies regarding MD of operation time

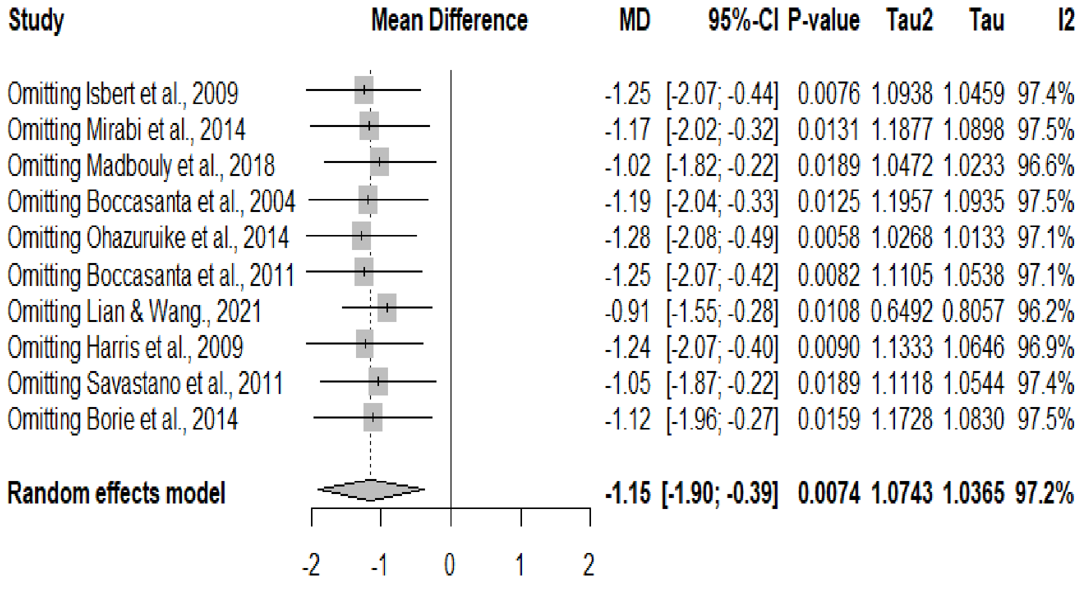

Figure S2K: Pooled effect (MD, CI) and heterogeneity I^2 of LOS after conducting leave one sensitivity analysis per study

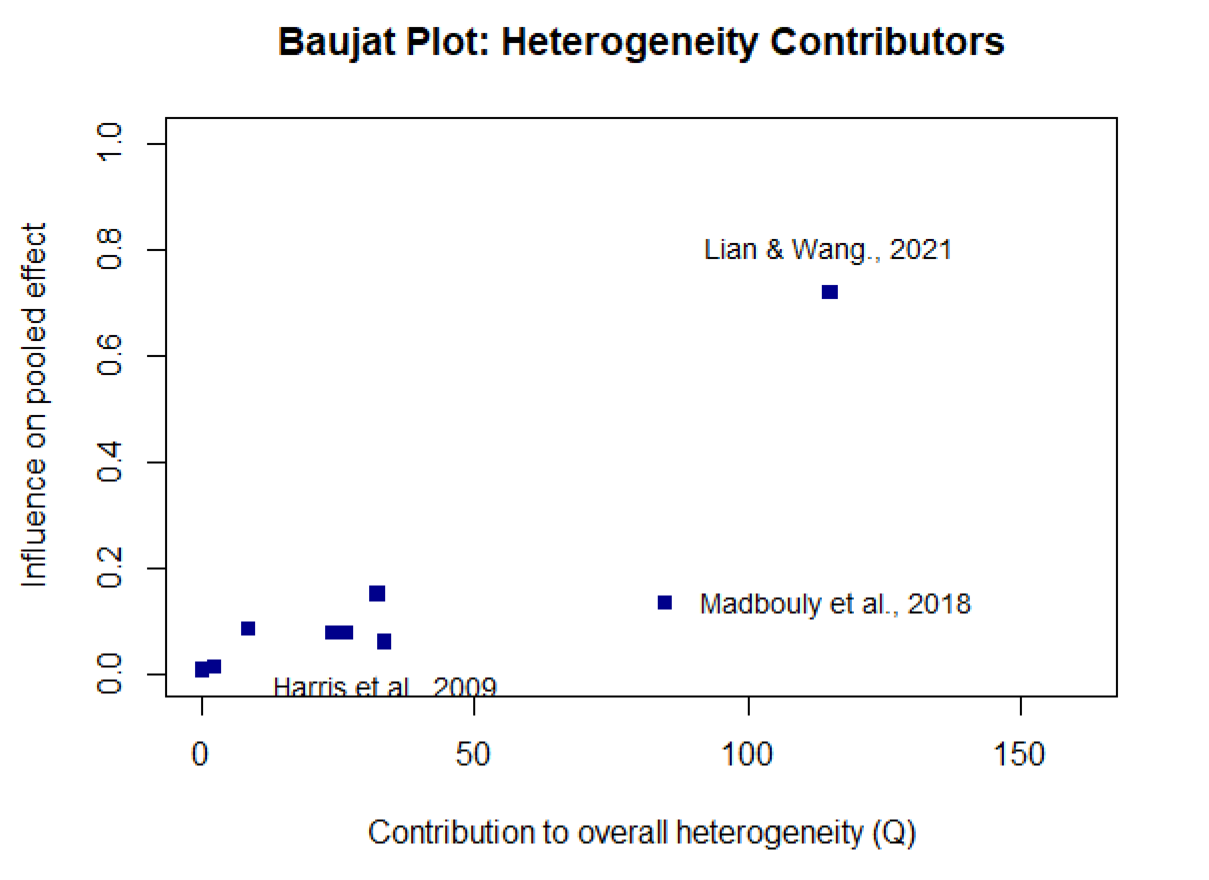

Figure S2L: Baujat plot for LOS checking for influential and outliers, showing contribution of each included study on the pooled effect and the overall heterogeneity

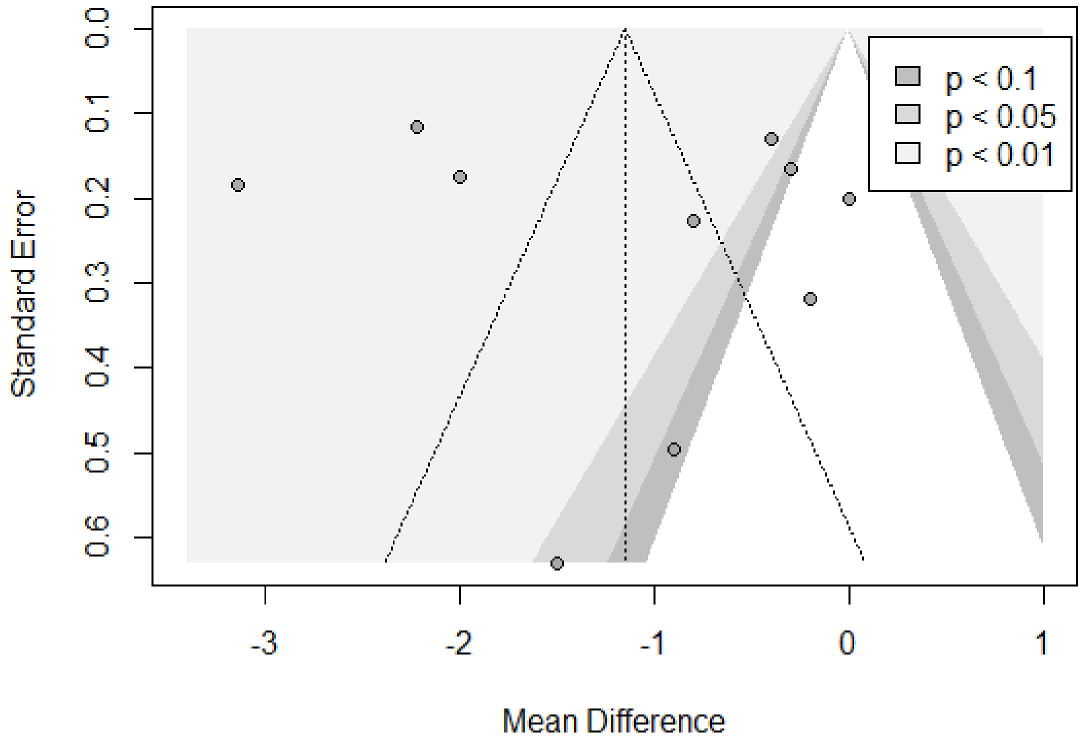

Figure S2M: Funnel plot of publication bias for published studies regarding MD of LOS
